# Metabolic profiling of bacteria with the application of polypyrrole-MOF SPME fibers and plasmonic nanostructured LDI-MS substrates

**DOI:** 10.1038/s41598-024-56107-0

**Published:** 2024-03-06

**Authors:** Radik Mametov, Gulyaim Sagandykova, Fernanda Monedeiro, Aleksandra Florkiewicz, Piotr Piszczek, Aleksandra Radtke, Pawel Pomastowski

**Affiliations:** 1 Centre for Modern Interdisciplinary Technologies, Nicolaus Copernicus University in Toruń, Wileńska 4, 87-100 Toruń, Poland; 2 Department of Chemistry, Faculty of Philosophy, Sciences and Letters of Ribeirão Preto, University of São Paulo, Av. Bandeirantes 3900, Ribeirão Preto, 14040-901 Brazil; 3 Department of Inorganic and Coordination Chemistry, Faculty of Chemistry, Nicolaus Copernicus University in Toruń, Gagarina 7, 87-100 Toruń, Poland

**Keywords:** Microbiology, Materials science, Chemistry, Analytical chemistry, Bioanalytical chemistry, Mass spectrometry

## Abstract

Here we present application of innovative lab-made analytical devices such as plasmonic silver nanostructured substrates and polypyrrole-MOF solid-phase microextraction fibers for metabolic profiling of bacteria. For the first time, comprehensive metabolic profiling of both volatile and non-volatile low-molecular weight compounds in eight bacterial strains was carried out with utilization of lab-made devices. Profiles of low molecular weight metabolites were analyzed for similarities and differences using principal component analysis, hierarchical cluster analysis and random forest algorithm. The results showed clear differentiation between Gram positive (G+) and Gram negative (G−) species which were identified as distinct clusters according to their volatile metabolites. In case of non-volatile metabolites, differentiation between G+ and G− species and clustering for all eight species were observed for the chloroform fraction of the Bligh & Dyer extract, while methanolic fraction failed to recover specific ions in the profile. Furthermore, the results showed correlation between volatile and non-volatile metabolites, which suggests that lab-made devices presented in the current study might be complementary and therefore, useful for species differentiation and gaining insights into bacterial metabolic pathways.

## Introduction

Infectious diseases pose a global health concern. Development of rapid and accurate identification methods plays a pivotal role in safeguarding public health.

Mass spectrometric platforms such as MALDI Biotyper^1^ and VITEK MS^2^ have been developed as efficient alternative for microbiological culturing with subsequent biochemical identification. The underlying principle of these platforms revolves around the profiling of bacterial membrane proteome and their comparison with a database with application of matrix-assisted laser desorption/ionization (MALDI). In addition to proteomic profiling, profiling of low molecular weight (LMW) volatile organic compounds and non-volatile metabolites has also received increased attention.

Bacterial membrane lipids possess rich structural diversity^3^, which can be useful in species differentiation. For the purpose of profiling of bacterial membrane lipids, application of laser desorption/ionization (LDI) techniques for profiling of membrane lipids offers advantage in terms of sensitivity, simplicity of use, low sample volume and relatively fast time of analysis.

Leung et al.^4^ reported that bacterial membrane glycolipids were specific for different clinically significant pathogens with application of MALDI coupled to time-of-flight mass spectrometry (TOF–MS). Similar approach was applied in the study reported by Liang et al.^5^ with the exception of sample preparation method, where authors proposed aqueous sodium acetate buffer for efficient extraction of lipids. The applied approach allowed for identification of four clinically relevant bacterial strains in < 1 h. Another LDI technique, promising for rapid profiling of bacterial membrane lipids, is nanomaterials-assisted LDI mass spectrometry (NALDI-MS), where nanomaterials are utilized for desorption and ionization of analytes in the sample^6^. NALDI demonstrated enhanced sensitivity towards various low molecular weight analytes including lipids^7^, especially with utilization of plasmonic nanostructures. Plasmonic LDI-MS substrates demonstrated utility for analysis of clinically relevant biomolecules^8,9^ owing to localized surface plasmon resonances (LSPRs) upon interaction of the nanomaterials with light. However, there have been no studies reporting the application of plasmonic LDI-MS substrates in attempt to differentiate between bacterial species.

Efforts to differentiate bacterial species utilizing profiling of volatile organic compounds (VOCs) have been in progress for more than three decades. Initial studies utilized bacterial VOCs collection techniques, like traps made from a sorbent material such as Tenax^10^. Nowadays, gold standard in the bacteria VOCs profile collection is headspace solid-phase microextraction (HS-SPME) coupled with gas chromatography (GC)^11^. SPME was introduced in 1990 and received wide recognition as a simple and solvent-free method^12^. Recent studies by Reese^13^ and Fitzgerald^14^ reported volatile profiles of various pathogenic bacteria, demonstrating potential of utility of VOCs profiling in genus- and species-level discrimination. It is worth mentioning that all of the mentioned works reported application of commercially available SPME fibers. Introduction of new materials as extraction coating might be helpful in revealing specific bacterial metabolites due to the changes in affinity and therefore, specificity of analysis. Numerous papers exploring the application of novel materials as extraction coating for SPME were reported in the literature^15^. However, there is a lack of studies reporting application of lab-made SPME fibers based on new materials as extraction coatings for detection and profiling of bacterial volatile metabolites.

While previous studies have extensively explored volatile or non-volatile metabolites, rare efforts were dedicated to metabolic profiling of bacteria with an emphasis on both groups aiming at species differentiation. To the best of our knowledge, only one study by Wang et al.^16^ has been carried out with utilization of silver nanostructured substrates for surface enhanced Raman spectroscopy to detect both volatile and non-volatile metabolites aiming at bacterial quantification and growth monitoring.

Hence, we introduce here alternative innovative lab-made analytical devices for metabolic profiling of bacteria. Analytical devices encompass polypyrrole-MOF (PPy@ZIF-8) HS-SPME fiber and plasmonic silver nanostructured LDI-MS substrates, and their simultaneous use allowed for comprehensive profiling of both volatile and non-volatile bacterial metabolites of eight strains of bacteria.

## Materials and methods

### Reagents and materials

All reagents and solvents used in the current study were of the highest available purity and purchased from Sigma Aldrich (Steinheim, Germany). Organic matrices for matrix-assisted laser desorption/ionization mass spectrometry (MALDI-MS) such as hydroxycinnamic acid (HCCA) and dihydroxybenzoic acid (DHB) were purchased from Bruker Daltonics (Bremen, Germany). Brain Heart Infusion Agar (BHIA) was purchased from Sigma Aldrich (Steinheim, Germany). Water was obtained using the Milli-Q RG apparatus by Millipore (Millipore Intertech, Bedford, MA, USA). Commercial SPME fiber, namely 75 μm carboxen/polydimethylsiloxane (CAR/PDMS) was purchased from Agilent Technologies, California, USA).

### Culturing of bacteria

Eight strains of bacteria, namely *Morganella morganii* (MM), *Staphylococcus warneri* (SW), *Lactobacillus plantarum* (LP), *Enterococcus faecium* (EF), *Enterococcus durans* (ED), *Lactococcus garvieae* (LG), *Staphylococcus epidermidis* (SE), and *Escherichia coli* (EC) were obtained from Microbank® cryovials (Pro-Lab Diagnostics, UK) deposited at −80 °C^17^ and grown using a modified method previously described by our research team^18^. In order to confirm the identification of selected species of microorganisms, one bead was inoculated on Petri dishes (Alchem, Poland) with solid Mueller Hinton Agar medium (Sigma Aldrich, Germany). A microbial loop (1 μL) of bacterial biomass was applied directly to the plate, according to the procedure recommended by the manufacturer. A further procedure consisting of a bacterial protein extraction protocol using microorganism identification analysis using the MALDI-TOF–MS technique and the MALDI Biotyper 3.0 platform (Bruker Daltonics, Bremen, Germany) has been described in previous works of our team^18,19^.

### Extraction of lipids

For collection of non-volatile metabolic profiles, extraction has been carried out using the standard Bligh & Dyer^20^ (B & D) method with modifications. In the attempt of sampling standardization, 100 mg of bacterial biomass was collected from five separate Petri dishes. After separation of chloroform and methanol phases, solvents were evaporated and 1.5-mL Eppendorf vials were weighted to determine the mass of the dry residue. Stock solution for each phase was prepared by addition of corresponding solvent (chloroform and methanol) in microliters to final ratio between the dry residue and solvent 1:1.

### Growth curves experiment

In order to determine the growth curves for the selected microorganisms, we transplanted fresh bacterial colonies that had been grown in MHA culture medium into glass tubes containing Mueller Hinton Broth (MHB). This process aimed to achieve a 0.50 McFarland (McF) standard, measured at a wavelength of λ = 565 ± 15 nm using a DEN-1B Densitometer, which operates based on turbidity approaches (Biosan, Józefów, Poland). For the McFarland 0.5 Standard, the approximate OD at 600 nm is between 0.08 and 0.13^21^. Additionally, measurements of optical density (OD) values at λ = 600 ± 1 nm were conducted using a Thermo Scientific™ Varioskan™ LUX, with further details provided in the supplementary data (Fig. S17). The control used in the experiment was MHB medium alone. The prepared bacterial suspensions were incubated at 37 °C under aerobic conditions, with measurements taken hourly for approximately 32 h. Each experiment was conducted with at least three replicates to ensure consistency and reliability.

### HS–SPME–GC–MS profiles

All bacteria species were inoculated and grown in disposable culture tubes with round-bottom and screw caps to enable measurement of concentration directly before the extraction of VOCs. Septa from disposable culture tubes were exchanged to silicone/PTFE 18 mm from the classical 20-mL headspace vials to prevent losses of volatile compounds. All tubes, septa, caps and medium were autoclaved for sterilization. For extraction of VOCs of bacteria, lab-made PPy@ZIF-8 coated SPME fiber was utilized.

Prior to performing the sample analysis, pre-conditioned fibers were exposed to the headspace of empty sterile culture tubes, and blank analyses were carried out. Furthermore, we also performed an analysis of the culture media used for bacteria inoculation. Any signals originating from blank analyses (potentially fiber material) and culture media were excluded from identification and were not considered. After each three runs the blank of the SPME fibers was taken to ensure the absence of contaminants.

For extraction of VOCs, the following parameters were used: equilibration time was dependent on certain types of bacteria species. Extraction temperature was set as 37 °C and time of extraction set as 40 min. The desorption process was performed in the GC-inlet at 220 °C for 5 min.

Gas chromatographic analyses of VOCs released by bacteria were conducted using a GC 7820A gas chromatograph coupled with an Agilent 5977B mass spectrometer MSD (Agilent Technologies, Waldbronn, Germany). The GC system was equipped with a ZB-624 capillary column (30 m × 0.25 mm × 1.4 μm). Helium was employed as the carrier gas, flowing continuously at a rate of 1 mL/min. Injections were carried out using the splitless mode, and the GC injector port was maintained at a temperature of 220 °C. HS–SPME–GC–MS profiles of all strains were collected with consideration of growth phases.

The temperature program for the GC oven initiated at 30 °C and was held for 4 min, after which it was ramped at a rate of 7 °C min^−1^ to reach 150 °C (maintained for 2 min). Subsequently, the temperature was raised to 250 °C at a rate of 10 °C min^−1^. The final oven temperature was held constant for 5 min. The mass spectrometer was operated in the electron impact (EI) mode with an energy of 70 eV. The ion source temperature and the transfer line temperature were set to 230 °C and 250 °C, respectively. Data acquisition was performed at a frequency of 2.9 scans per second, covering a mass range of 35 to 550 atomic mass units (a.m.u.). Compound identification was processed by searching the obtained mass spectrum in the NIST11 mass spectral library. The criterion for peak detection was a signal-to-noise of at least 3, and peak integration was performed manually. Spectrum search encompassed baseline subtraction and averaging over a peak. Each peak was searched manually, including baseline subtraction and averaging over a peak. Forward match quality of at least 700/1000 was applied as the lower match threshold. Peaks detected in samples corresponding to pure culture media were deleted from the total dataset, for the obtainment of signals attributed solely to bacteria sample. Chromatographic data was processed using the software MassHunter Qualitative analysis 10.0. Signal integration step was based on the total ion current (TIC) of the peak, as the employed methodology set-up was ideal for an optimized separation of the compounds of interest, only significant peaks were considered, and deconvolution procedures were not performed. A table containing bacterial VOCs identified in each culture is provided in the Supplementary Material (Table S1). To ensure reliability of identification, it has been carried out with consideration of several parameters, such as probability of match (minimal threshold was set to 75%), retention index and retention time, peak shape and spectra compared to a reference standard.

For comparison of extraction performance of PPy@ZIF-8 and commercial carboxen/polydimethylsiloxane (CAR/PDMS) fibers, standard solution of compounds with distinct structures was prepared. The solution was prepared in methanol with the final concentration 17 µg/mL for each analyte. Commercial fiber was pre-conditioned prior to extraction, following the guidelines of the manufacturer. Extraction has been carried out at 30 °C for 49 min with stirring 750 rpm. The solution was pre-incubated for 17 min at room temperature. The sample volume accounted for 3 mL. 1 g of NaCl was added to solution prior to extraction. GC analyses of standard solution after extraction were carried out with a gas chromatograph GC 7820A coupled to a flame ionization detector (Agilent, Santa Clara, CA, USA). The instrument was equipped with HP-5 analytical column (30 m × 0.32 mm i. d. and film thickness 0.25 μm). Carrier and make-up gases were helium (99.99%) and air. Analyses were performed in the splitless mode, injector port operated at 220 °C. Detector operated at 300 °C and the carrier gas flow rate was 2.4 mL/min. The initial oven temperature was 30 °C (held for 3 min), ramped at a rate of 4 °C min^−1^ to 50 °C (held for 1 min), then increased to 70, 100 (held for 1 min), and 200 °C at the rates of 5 °C min^−1^, 7 °C min^−1^ and 40 °C min^−1^, respectively. The final oven temperature was kept for 3 min. Make-up gas, hydrogen, and synthetic air flow were maintained at 30 mL/min, 30 mL/min, and 300 mL/min, respectively.

### NALDI-MS profiles

NALDI-MS profiles of bacterial extracts were collected using silver nanostructured substrates with size of nanoparticles 50 ± 10 nm^22^, and organic matrices (HCCA and DHB) as a reference technique. In case of MALDI, organic matrices were prepared at concentration 10 mg/mL in TA30 solution. Deposition of samples was performed using dried droplet technique: 2.5 µL of matrix solution was mixed with 2.5 µL of sample, and 1 µL was deposited onto the target plate (ground steel target, Bruker Daltonics, Bremen, Germany).

To consider the differences in concentration of lipids, stock solutions and dilutions (10, 10^2^ and 10^3^ times) were analyzed using MALDI and NALDI to determine the dilution providing with intense signals in the lipid region (*m/z* 400–1500) in MS spectra. For MALDI, all bacterial extracts were analyzed using the stock solution. In case of NALDI, all chloroform extracts were registered using dilution factor 10, except for *Enterococcus Faecium*, where stock solution was used, and stock solution was used in case of methanol fractions.

For both MALDI and NALDI, UltraFlextreme MALDI-TOF–MS instrument (Bruker Daltonics, Bremen, Germany) with a modified neodymium-doped yttrium aluminium garnet (Nd:YAG) laser operating at 355 nm and frequency 2 kHz was used. The profiles were collected in ion-positive mode in the mass range *m/z* 60–1400 and the number of shots for single MS spectrum collection accounted for 2500. Mass calibration for MALDI analyses was carried out using clusters of CsI_3_: 2.5 µL of 10 mg/mL solution of CsI_3_ in methanol was mixed to 2.5 µL of 10 mg/mL of DHB matrix in methanol and 1 µL was deposited onto the target plate. In case of NALDI, 1 µL of extracts were deposited directly onto the solid substrate and internal mass calibration using signals of silver was carried out. For all LDI measurements, cubic enhanced calibration method was used. For NALDI, the following instrument parameters were applied: 80% of laser power, detector gain 30× , value of global attenuator of 30% and parameter set ‘ultra’. For MALDI, the same parameters were used except for detector gain value, which was set to 2.51× in case of DHB. Profiles of NALDI were collected in 5 replications. For silver nanostructured substrates, MTP slide adapter II was used (Bruker Daltonics, Bremen, Germany). The list of all detected signals is provided in the Supplementary Material (Tables S2–S17).

### Data analysis

Data analysis was conducted in R environment (R v.4.2.1), using RStudio console (v. 2022.02.03, PBC, Boston, MA, USA). Principal component analysis (PCA) was performed using the packages “factoextra” and “FactoMineR”. Heatmaps (“pheatmap” package) used as input the scaled average values of peak area and ion intensity, in case of VOCs and LDI-MS data, respectively. For hierarchical clustering analysis (HCA), Euclidean distance was used to measure the association between samples, and Ward’s was selected as the clustering method. Normality of data distribution was verified using Shapiro–Wilk test (“stats” package). Statistical comparison between ion intensities obtained from LDI-MS using different extraction phases was carried out using Mann–Whitney test (“stats” package). Random forest (RF) model was created using “randomForest” package, employing the following parameters: number of trees = 900, number of variables randomly sampled as candidates at each split = 3, cut-off = 1/k (majority vote wins, where k is the number of classes, i.e., 8). After a random split, 60% of the data was used for model training, while the remaining 40% was employed for model testing. Canonical correlation analysis (CCA) was carried out using “vegan” package, while univariate correlation analysis (Spearman’s method) was conducted using “Hmisc”. Networks were built with the aid of “igraph” and “visNetwork” packages.

## Results and discussion

### Bacteria growth curves

To collect volatile metabolites, we carried out investigation of the growth phases for all strains.

**Figure 1:**
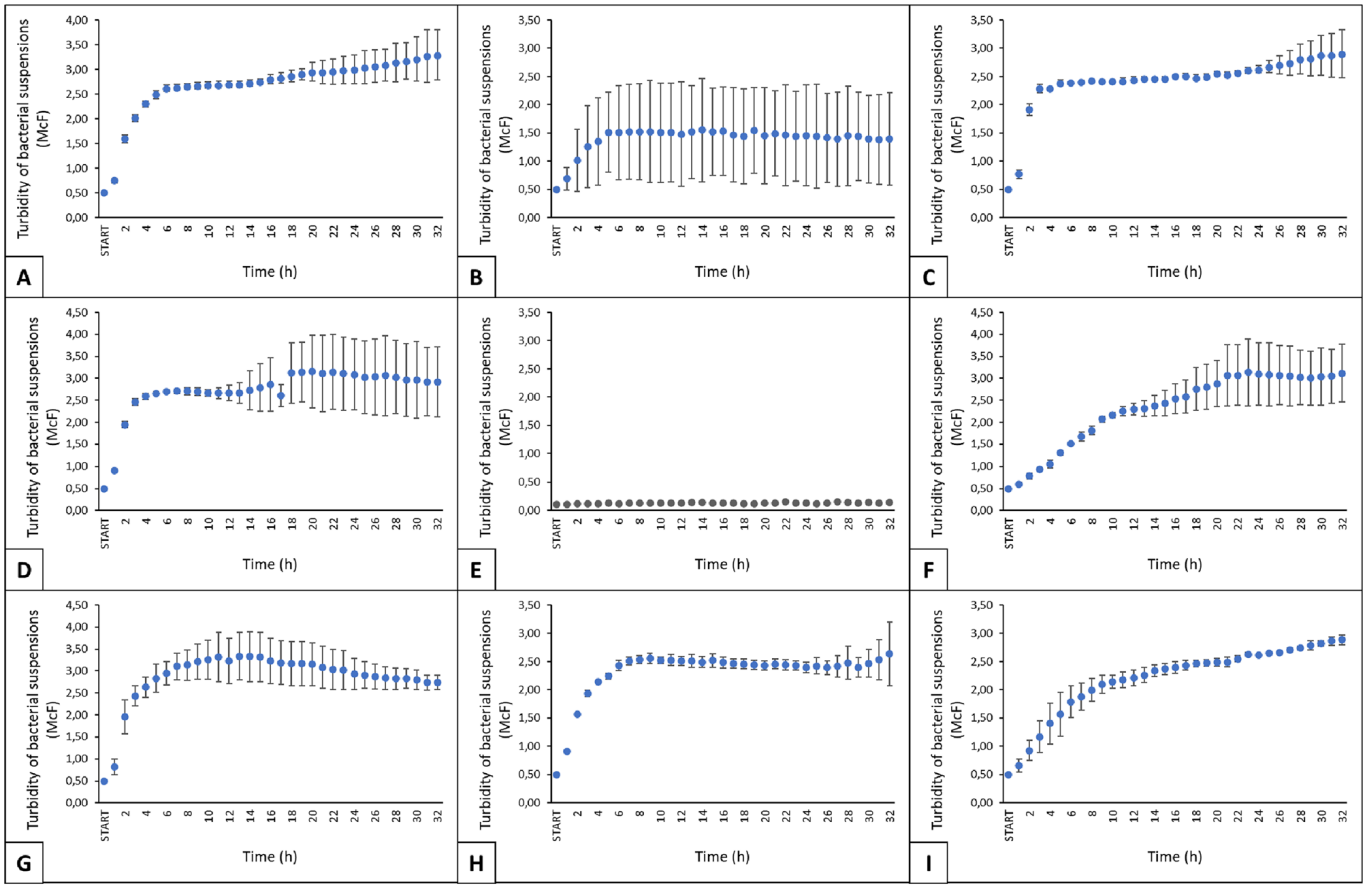
Microbial growth curves of bacteria grown at 37 °C for 32h. (**a**) *Escherichia coli*, (**b**) *Lactobacillus plantarum*, (**c**) *Morganella morganii*, (**d**) *Enterococcus durans*, (**d**) control, Mueller Hinton Broth medium, (**f**) *Staphylococcus epidermidis*, (**g**) *Enterococcus faecium*, (**h**) *Lactococcus garvieae*, (**i**) *Staphylococcus warneri*.

The growth curves of selected microorganisms as a function of time are shown in Fig. 1. All isolated microorganism strains showed good ability to grow in MRS broth at 37 °C. For the temperature 37 °C, the exponential growth phase was observed between 10 and 16 h (Fig. 1).

Bacterial suspension turbidity (McF) values are shown as averages of three technical replicates ± SE. For *E. coli* cultured at 37 °C, there was an apparent exponential growth phase between 2 and 8 h, seemingly reaching a stationary phase between 6 and 12 h (Fig. 1A). Under ideal conditions, E. coli in a rich liquid broth medium is speculated to have a doubling time of approximately 20 min and may reach a cell density greater than 10^9^ CFU/mL after an overnight culture^23,24^. Similarly, *L. plantarum* at 37 °C appeared to enter exponential growth between 2 and 8 h, with a presumed stationary phase from 5 to 12 h (Fig. 1B). A comparable pattern at both 37 °C and 45 °C was suggested in the study by Smetanková et al.^25^. The growth variability of *Lactobacillus plantarum*, distinct from other bacteria, is attributed to its unique nutritional needs and heightened sensitivity to environmental factors like oxygen levels, temperature, and pH. Its specific metabolic pathway, fermenting sugars into lactic acid, further contributes to this variability, especially in standard laboratory settings. Understanding these unique requirements is key to optimizing L. plantarum cultivation and offers insights into bacterial growth dynamics under varied conditions^26^.

In turn, the bacterium *M. morganii* (Fig. 1C) grew exponentially up to 5 h of culture and reached stationary phase between 6 and 10 h. This bacterium is known^27^ to grow in the temperature range from 4 to 45 °C, identical growth of this bacterium for 37°C was shown by the results of Minnullina et al.^28^. In the case of *E. durans* (Fig. 1D), the exponential growth phase possibly lasted up to 6 h, and a stationary phase might have occurred from 10 to 14 h. The control of the experiment, MHB medium, showed no traditional growth curve (Fig. 1E). *S. epidermidis* demonstrated specific proliferation patterns (Fig. 1F), inferred from the growth curve where the exponential phase lasted around 20 h, and a stationary phase, with minor fluctuations, was likely reached between 20 and 32 h. This is confirmed by a study by Mantripragada et al.^29^, in which the exponential growth phase of *S. epidermidis* lasted up to 9 h of incubation, but the typical plateau phase was not characterized, although bacterial proliferation was no longer as intense. *E. faecium*, as shown in Fig. 1G, appeared to reach exponential phase between 2 and 8 h, and a stationary phase between 13 and 15 h, followed by a gradual decline. In a study by Zhang et al.^30^, the growth curves of E. faecium strain E1162 and various mutants indicated exponential growth up to 5 h, with a plateau phase from 5 to 9 h, under incubation at 37 °C in BHI medium with ampicillin^30^. *L. garvieae* exhibited a probable exponential growth up to 9 h, and a plateau phase from 10 to 15 h. In the study by Xie et al.^31^, the growth curve of *L. garvieae* was characterized by an exponential phase reached by 6 h of incubation at 37 °C in BHI medium, while the stationary phase was reached between 10 and 16 h of culture. Finally, S. warneri (Fig. 1I) seemed to reach an exponential phase by 10 h of incubation, and while a typical stationary phase was not clearly observed, it is speculated to be between 18 and 20 h. Hourly observations in TSB medium suggested that the final logarithmic or exponential phase of S. warneri MBF02-19J lasted for 17 h^32^.

### Investigation of patterns within volatile and non-volatile LMW profiles

**Figure 2:**
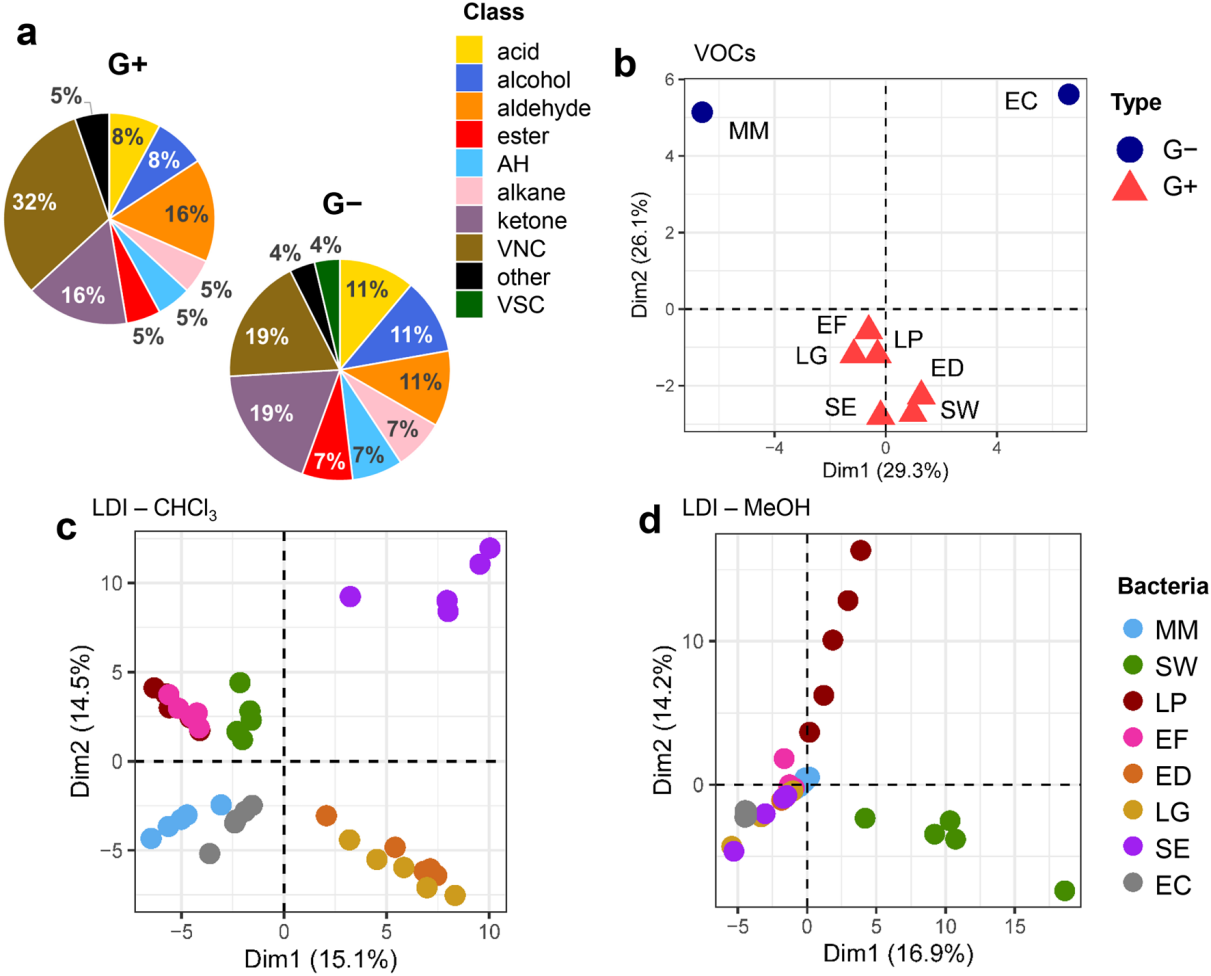
(**a**) Fractions of each VOC class detected among G+ and G− bacteria; PCA score plots for (**b**) VOCs data and NALDI-MS data, considering the analysis of (**c**) the chloroform phase and (**d**) the methanol phase (n = 5 per bacteria). *AH* aromatic hydrocarbon, *VNC* volatile nitrogen-containing compound, *VSC* volatile sulfur-containing compound.

The PPy@ZIF8 SPME fiber allowed the recovery of a total of 68 different VOCs, associated with bacteria presence in the cultures. From these, 40 VOCs could be annotated according with the compound identification criteria. Each different bacterial volatile detected across the cultures was attributed to a VOC class, and the number of unique VOCs ascribed to a given class was evaluated (in terms of percentage) in relation to the total number of bacterial VOCs (100%) (Fig. 2a, b).

**Figure 3:**
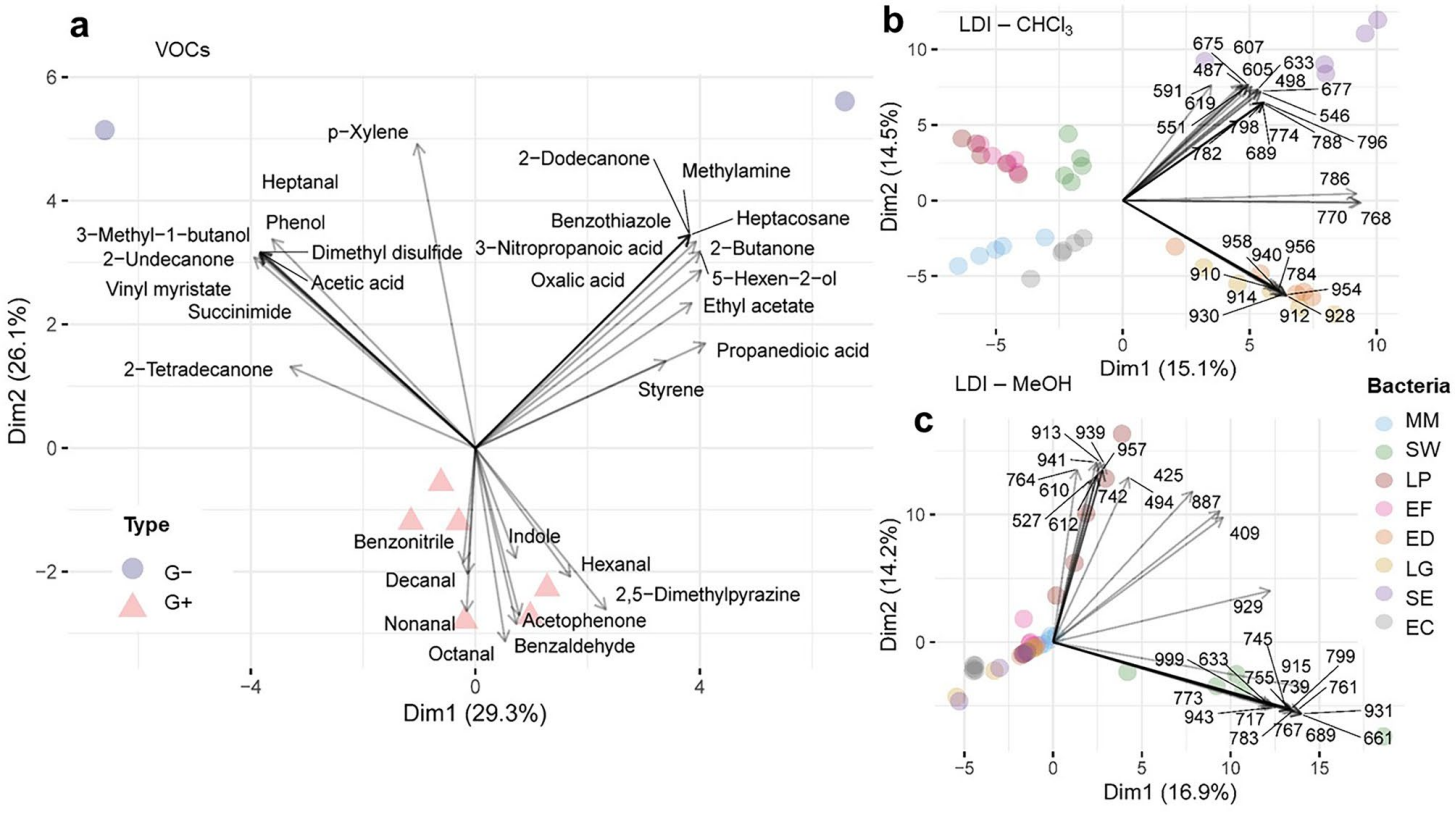
PCA variable plots displaying the top 30 most contributing variables, for (**a**) VOCs data, as well as NALDI-MS data obtained from the analysis of (**b**) chloroform and (**c**) methanol phases (n = 5 per bacteria).

Identified VOCs recovered from bacterial cultures consisted mainly of volatile nitrogen-containing compounds (VNCs, 19–32%), followed by ketones (16–19%), aldehydes (11–16%), alcohols and organic acids (8–11%). In the attempt of comparison of our data for selected compounds extracted by PPy@ZIF-8 and data reported in the literature, the following observation has been made. The previous study^33^, conducted by our research group, stressed VOCs emitted by various strains, including *Staphylococcus warneri*, with utilization of the PDMS/DVB commercial fiber. In this study^33^, the predominant group of detected compounds were ketones, hydrocarbons and alcohols (Fig. 3C)^33^. In our study, predominant group of compounds was nitrogen-containing compounds in case of *Staphylococcus warneri* and other species. Therefore, the affinity of PPy@ZIF-8 fiber towards nitrogen-containing compounds has been suggested, especially for the pyrrole-ring containing compounds. On the other hand, Drabińska and co-authors^34^ performed the experiment for extraction of VOCs by SPME–GC–MS/MOS method and utilized CAR/PDMS commercial fiber. According to the results, detected compounds included benzaldehydes, pyrazines derivatives, xylene etc^34^, and some of the compounds were similar as in the current study. In addition, it is essential to consider that the composition of VOCs’ profiles depend on strain and sample matrix, culture media and growth conditions. Hence, we propose that the prevalence of nitrogen-containing compounds in the profiles may not necessarily be linked to the affinity toward nitrogen within the polypyrrole ring's structure. Moreover, the efficiency of SPME fiber in a headspace mode is a complex process influenced by various factors. The interplay between fiber properties and the chemical structures of analytes becomes particularly significant in chemically-rich sample matrices^35^, such as bacteria. To exemplify the diversity of responses in complex mixtures, we examined the extraction performance of both PPy@ZIF-8 and commercial CAR/PDMS fibers using a standard mixture of compounds with distinct chemical structures (Table S18). The results indicated that the CAR/PDMS coating is the mostly non-polar, while the PPY@ZIF-8 coating is conversely more polar. Interestingly, despite non-polar nature of dodecane, PPY@ZIF-8 exhibited a high response, likely due to the affinity of polypyrrole to long-chain linear hydrocarbons^36^. Lab-made coating demonstrated approximately two times lower efficiency for extracting BTEX group analytes compared to CAR/PDMS, suggesting a weaker affinity of lab-made SPME fibers for non-polar analytes. This may be attributed to competition on the fiber, where binding sites was occupied by more polar compounds such as methanol.

Since many biologically active bacterial metabolites are VNCs (e.g., indole, pyrazines), the prepared fiber might be favorable for the study of microbial interactions. Here, such greater recovery of VNCs is possibly attributed to the affinity of VNCs towards nitrogen in the structure of PPy@ZIF-8 coating^17^ as well as the unequal distribution of electrons leading to dipole moment. Differences were observed regarding the fraction of VOC classes prevalent in the headspace of bacteria, depending on if these were G+ or G− species (Fig. 2a). G+ presented a greater proportion of VNCs and aldehydes while G−, while G− displayed a greater percentage of ketones, alcohols and acids. Besides, dimethyl disulfide—a volatile sulfur-containing compound (VSC), was detected only in G− species. PCA score plot of VOCs data (Fig. 2b) shows a clear discrimination between G+ and G− profiles, provided by the first PC. Although very distinct from G+ , VOC profiles from G− species also presented substantial differences between themselves, being discriminated by the second PC.

PCA was also used to explore patterns related with bacterial species within the NALDI-MS datasets. Figure 2c refers to lipid ions recovered from the chloroform fraction—in this case, each experimental replicate was plotted close to each other, indicating that the performed assays displayed adequate reproducibility. Reproducibility of profiles possibly was achieved due to nearly homogeneous distribution of silver nanoparticles^22^ acquired via chemical vapor deposition as compared to wet chemical synthesis, where coffee ring effect^37^ can lead to formation of hot spots and profiles inconsistency.

Moreover, clusters referring to individual species of bacteria can be observed, although an overlap between LP and EF, as well as between ED and LG is present. This overlap indicates a greater similarity between the lipid composition of these pair of bacteria. It is also of notice that the two G- bacteria (MM and EC) appear confined in the third quadrant of the plot. On the other hand, NALDI-MS lipid profiles acquired in the methanolic fraction for the same bacteria were not so congruent, displaying considerable intra-variability (Fig. 2d). Furthermore, the formed PCA clusters did not characterize most of bacteria species, with only LP and SW appearing as more distinct from others.

Figure 3 shows PCA variable plots, allowing us to verify which VOCs or lipid ions are correlated with the main PCs and the previously observed clusters. In these plots, only the top 30 variables contributing the most for data variance are showed. In case of VOCs (Fig. 3a), it was shown that G+ were characterized by an increased production of fatty aldehydes (hexanal, nonanal, decanal), benzaldehyde, acetophenone, 2,5-dimethylpyrazine and indole. According to literature, nitrogen-containing compounds, including pyrroles, and derivatives of pyrazine (such as 2,5-dimethylpyrazine, 2-ethyl-3,5-dimethylpyrazine, and 3-iso-pentyl-2,5-dimethylpyrazine), can be emitted by various bacterial strains^33^. Pyrazines, in particular, are noted as a prevalent group of compounds released by bacteria, although the metabolic pathways and biosynthesis are not fully elucidated^38^. Although *p*-xylene is a volatile compound considerably abundant in the indoor air, it has been also detected in bacterial cultures^39^. Such aromatic volatiles are possibly derived from intermediates of the shikimate pathway^40^. However, the analysis of blank sample of empty vial showed the absence of *p*-xylene as well as in the blank samples of media. Therefore, it was suggested that *p*-xylene could be emitted by bacteria. Nevertheless, the interpretation of data regarding the profiles of VOCs emitted by bacteria should be approached with caution. The existing literature suggests that pyrazines may be inherent to bacterial metabolism, but their presence can also arise from interactions between compounds in culturing media and bacterial metabolism, as well as chemical reactions occurring during the autoclaving of the media. Adams and Kimpe reported that standard test showed formation of pyrazine during autoclaving upon alkalization to pH 9 or higher suggesting that lysine that was added to the media served as a precursor for chemical formation of pyrazine by Maillard reaction^41^. Formation of pyrazines was also reported earlier by DeMilo et al.^42^, where authors concluded that their formation did not seem affected by bacterial action, but almost exclusively was affected by autoclaving of broth.

In the current investigation, commercial culturing media were employed, potentially accounting for the absence of pyrazines in the volatile profile of the media. This observation underscores the significance of meticulous consideration in the sample preparation of culturing media to investigate potential artifacts. Such considerations are pivotal for ensuring robust data interpretation in studies focused on bacterial metabolism. 

Regarding the cultures of G- bacteria, MM presented increased levels of 3-methyl-1-butanol, 2-undecanone, phenol and dimethyl sulfide. In contrast, EC cultures displayed augmented 2-dodecanone, benzothiazole, methylamine and heptacosane. In NALDI-MS data from the chloroform fraction (Fig. 3b), SE were marked by the increased response of lipids ranging from *m/z* 487 to 798, which in turn were depleted among G- species. LG and ED presented greater responses of the lipids corresponding to signals at *m/z* 784 and *m/z* 910–958, which were decreased among EF, LP, SW. In NALDI-MS data from methanolic phase (Fig. 3c), LP displays increased intensities for the lipid ions at *m/z* 913, 939, 941 and 957, among others. For SW, the lipids at *m/z* 661, 689, 767 and 931 were those which presented higher intensities. Conversely, the remaining bacterial species were characterized by decreased responses of the same lipid ions which were enhanced in LP profile.

**Figure 4:**
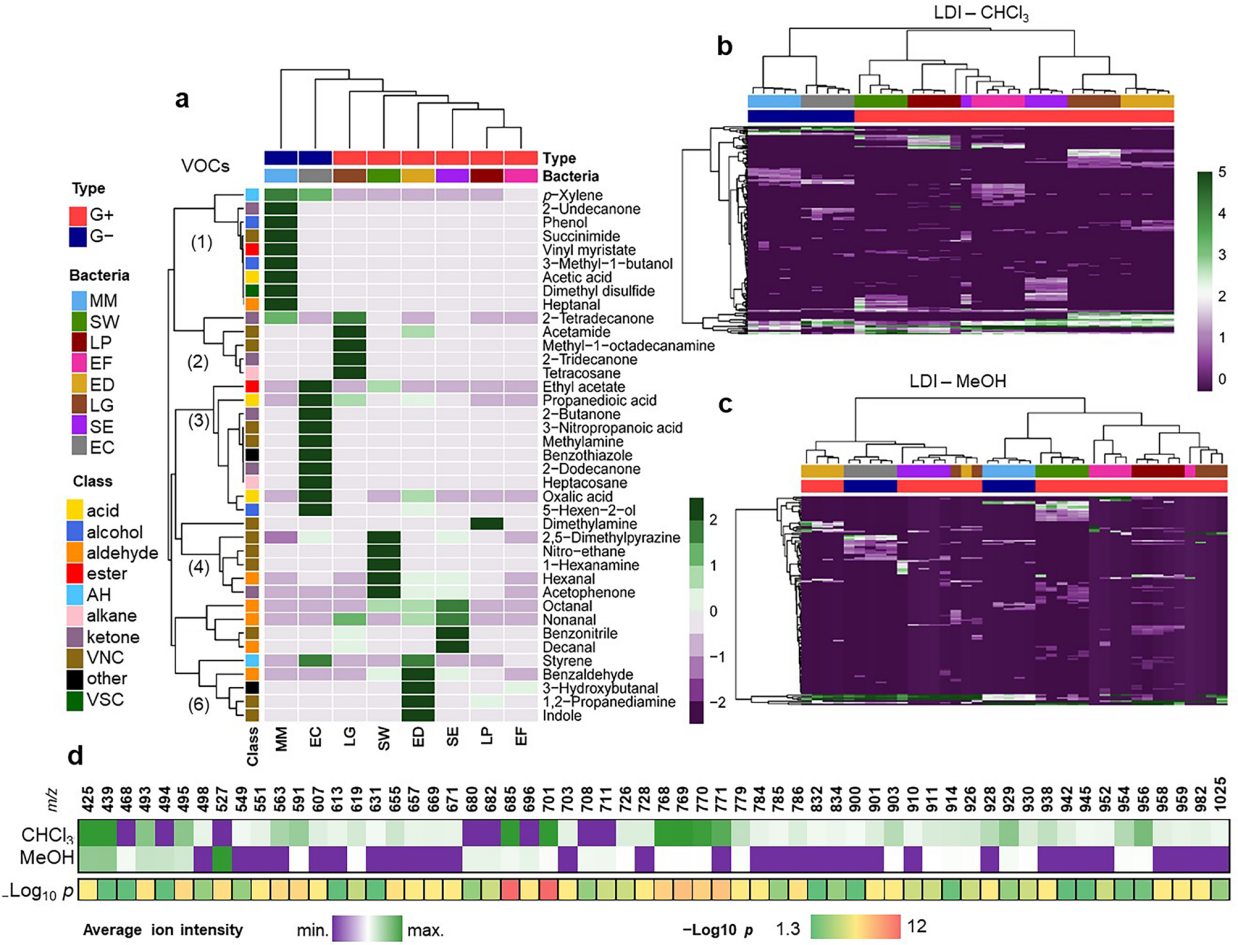
Hierarchical cluster analysis and associated heatmaps for (**a**) VOCs data, as well as NALDI-MS data obtained from the analysis of (**b**) chloroform and (**c**) methanol phases (n = 5 per bacteria); (**d**) chart showing the lipid ions which presented statistically significant differences in their intensities (*p* < 0.05), when comparing chloroform and methanol phases.

Correspondence between molecular profiles were also investigated using HCA. In this case, dissimilarities between samples were calculated based on the Euclidean distance. As observed in PCA, VOCs profiles obtained for G+ and G− bacteria were once more clustered separately (Fig. 4a). EC were the G− species more similar to G+, while EF appeared to be the most singular bacteria among other G+ species. Among VOCs, 6 clusters were identified based on the trends presented by these compounds across the samples of bacterial cultures. Clusters 1 (composed by various VOC classes) and 3 (mainly acids, ketones and VNCs) defined G+ species. The cluster 2 (ketones and VNCs) characterizes LG, while the clusters 4, 5 and 6 (mostly VNCs and aldehydes) define SW, ED and SE. Lastly, LP and EF were not particularly enriched with any of these main clusters. In NALDI-MS using the chloroform fraction (Fig. 4b), clusters corresponding to each bacterial species are observed, in addition to a clear distinction between G− and G+ bacteria. An overlap between species was only detected in case of SE and EF, the same bacteria which also presented a greater intersection in the corresponding PCA plots. On the other hand, lipid profiles obtained using the methanolic fraction did not display such coherent clustering of species (Fig. 4c). According to this approach, LG, EF and ED were the bacteria presenting less consistent lipid profiles.

NALDI-MS profiles of lipids obtained using the chloroform and methanol fractions were compared (Fig. 4d). The vast majority of ions presenting statistically significant differences in their intensities were better recovered in chloroform. Only signals at *m/z* 468, 494, 527, 680, 682, 696, 708 and 711 were uniquely detected in the methanolic phase—possibly, these are lipids with a stronger hydrophilic pole. This reasserts the results of the previous unsupervised analyzes, which demonstrated that chloroform fraction is the most suitable for the obtaining of representative and reproducible lipid profiles. Failure of differentiation between species based on methanolic fraction of the B & D extract could be caused by fragmentation of lipids in NALDI-MS with application of silver nanostructured substrates^22^. Our previous investigation demonstrated that plasmonic silver substrates exhibited sensitivity to various lipid classes^22^, which might be favorable for distinguishing between bacterial species due to the diversity of membrane lipids^3^. Nevertheless, fragmentation of phospholipids, especially polar phosphatidylcholine was observed^22^, which could potentially be attributed to photocatalytic properties of silver. This is in agreement with B & D extraction method, where polar lipids are extracted into the methanolic phase^43^. On the other hand, this also could be attributed to differences in the lipid content depending on the species. In addition, both MALDI and NALDI profiles showed less signals in the lipid region of the methanolic fraction of B & D extracts (Figs. S1–S8) as compared to chloroform phase (Figs. S9–S16). This aligns with previous investigation of our research group^44^, where silver nanostructured substrates enabled classification of the *Escherichia coli* strains into cefotaxime-resistant and cefotaxime-sensitive strains, while signals attributed to organic matrix hampered classification in case of application of MALDI.

The list of *m/z* values and corresponding intensities for the signals detected in the lipids’ mass region has been listed in Tables S2–S17. Since the current study was aimed at untargeted approach, and diversity of possible lipids, identification of the signals has not been performed. Nevertheless, the values of *m/z* could be compared with other data with assistance of the Lipid Maps database, which has been extended including non-mammalian sources of lipids^45^. Furthermore, due to extreme complexity of the sample matrix, identification of signals requires application of a targeted approach with a set of instrumental analytical techniques, including LC–MS with high resolution and opportunity to perform MS/MS analysis to generate specific fragments. For example, Oursel^46^ and co-authors investigated the lipid composition of *Escherichia coli* membranes using LC–ESI–MS/MS. However, the authors identified only phospholipids species, which can be explained by suitability of electrospray ionization for polar lipids^47^. Nevertheless, Jaber^48^ et al. reported di- and triacylglycerols detected in the lipidomic extract of *Escherichia coli* strains, however the precise data about identified species was not reported.

### RF model aiming for bacteria classification

**Figure 5:**
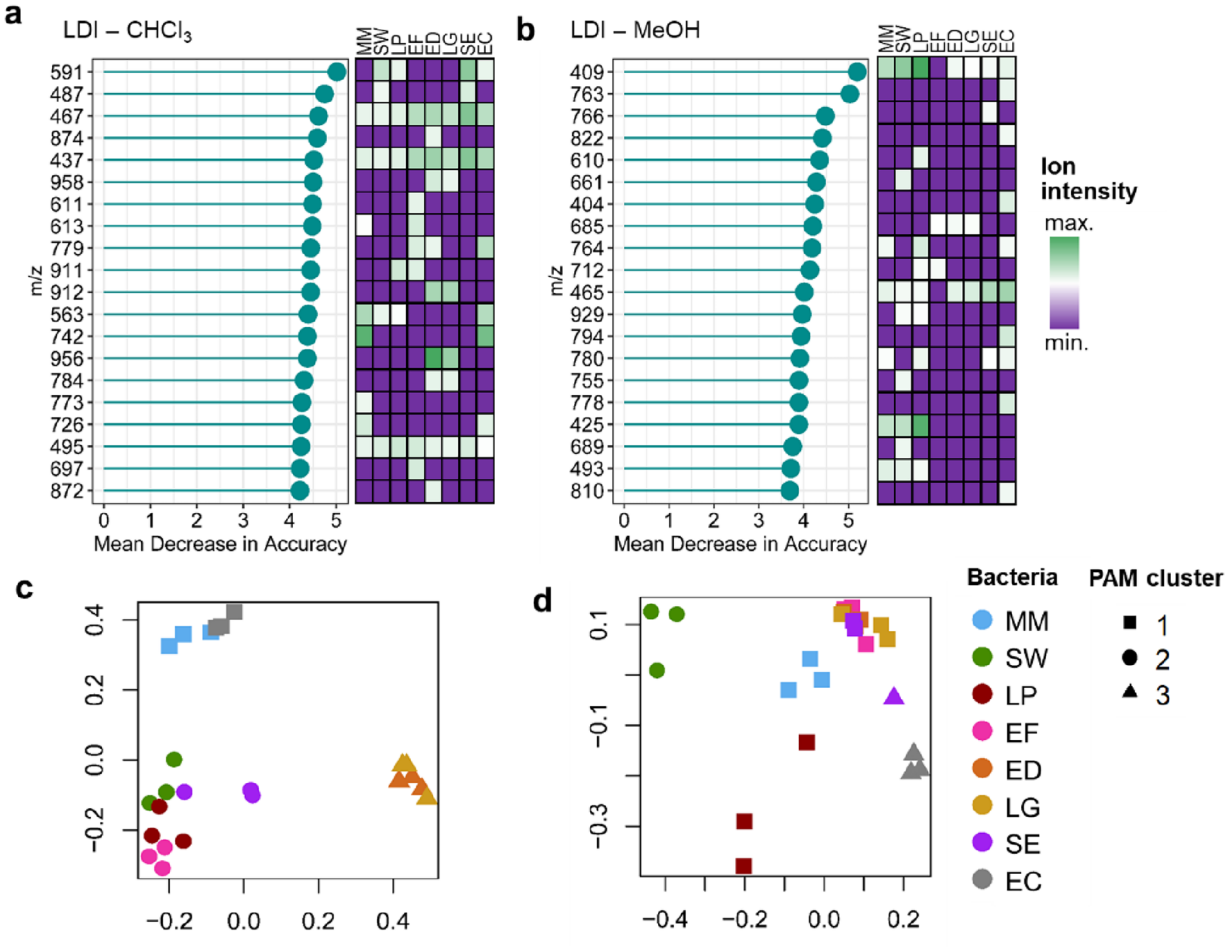
Variable importance rank in terms of mean decrease in accuracy, obtained according with RF model for LDI-MS in (**a**) chloroform and (**b**) methanol fractions; MDS plots built based on RF proximity matrix, applied to the calibration sets of LDI-MS data obtained from the analysis of (**c**) chloroform and (**d**) methanol phases.

Next, a model using RF algorithm was prepared, with the objective to classify bacterial species based on their lipid profiles obtained through NALDI-MS. The top 20 variables contributing the most for model accuracy are showed in Fig. 5a, b. These can be interpreted as the ions with the most distinctive responses across species, some of them being consistently unique for a given bacteria. Multidimensional scaling (MDS) plots of RF proximity matrices indicate the level of similarity between the questioned classes (i.e., bacteria species) according to model calibration (Fig. 5c, d). A greater distance between points correlates with a higher dissimilarity between samples. Therefore, a greater proximity between the points indicates species more prone to misclassification in the model. Partition around medoids (PAM) clustering allowed the classification of samples used for calibration as members of three different clusters. For example, in the chloroform fraction (Fig. 5c), G- species display a greater distance from the remaining species. Additionally, ED and LG appear grouped very closely, indicating the correspondence between these bacteria regarding their lipid composition. Analogous conclusions were made based on PCA results. Table 1 summarize the information regarding model performance. The out-of-bag (OOB) estimate of error rate obtained for NALDI-MS from chloroform and methanolic phases were 8.33% and 58.4%, respectively. As expected, NALDI-MS from the chloroform phase provided a superior balanced accuracy in the testing step (100%, 95%CI [79.4, 100%]). The excellent accuracy obtained for this dataset highlights the usefulness of NALDI-MS lipidomics for differentiation between bacterial species. For NALDI-MS of the methanolic fraction, balanced accuracy in the testing step was 75.0% (95%CI [47.6, 92.7%]). Lipid profiles from methanol phase failed to correctly classify the species EF, ED, LG and SE, the same appearing superimposed in the correspondent MDS plot.

**Table 1:** RF model performance (CI = confidence interval).

Phase	Parameter Bacteria	MM (%)	SW (%)	LP (%)	EF (%)	ED (%)	LG (%)	SE (%)	EC (%)
CHCl_3_	Sensitivity	100	100	100	100	100	100	100	100
	Specificity	100	100	100	100	100	100	100	100
	Balanced Accuracy	100	100	100	100	100	100	100	100
	Overall accuracy	100.0							
	95% CI	79.4–100							
MeOH	Sensitivity	100.0	100.0	100.0	50.0	100.0	0.0	50.0	100.0
	Specificity	100.0	100.0	100.0	85.7	85.7	100.0	100.0	100.0
	Balanced Accuracy	100.0	100.0	100.0	67.9	92.9	50.0	75.0	100.0
	Overall accuracy	75.0							
	95% CI	47.6–92.7							

### Correlations between volatile and non-volatile profiles

**Figure 6:**
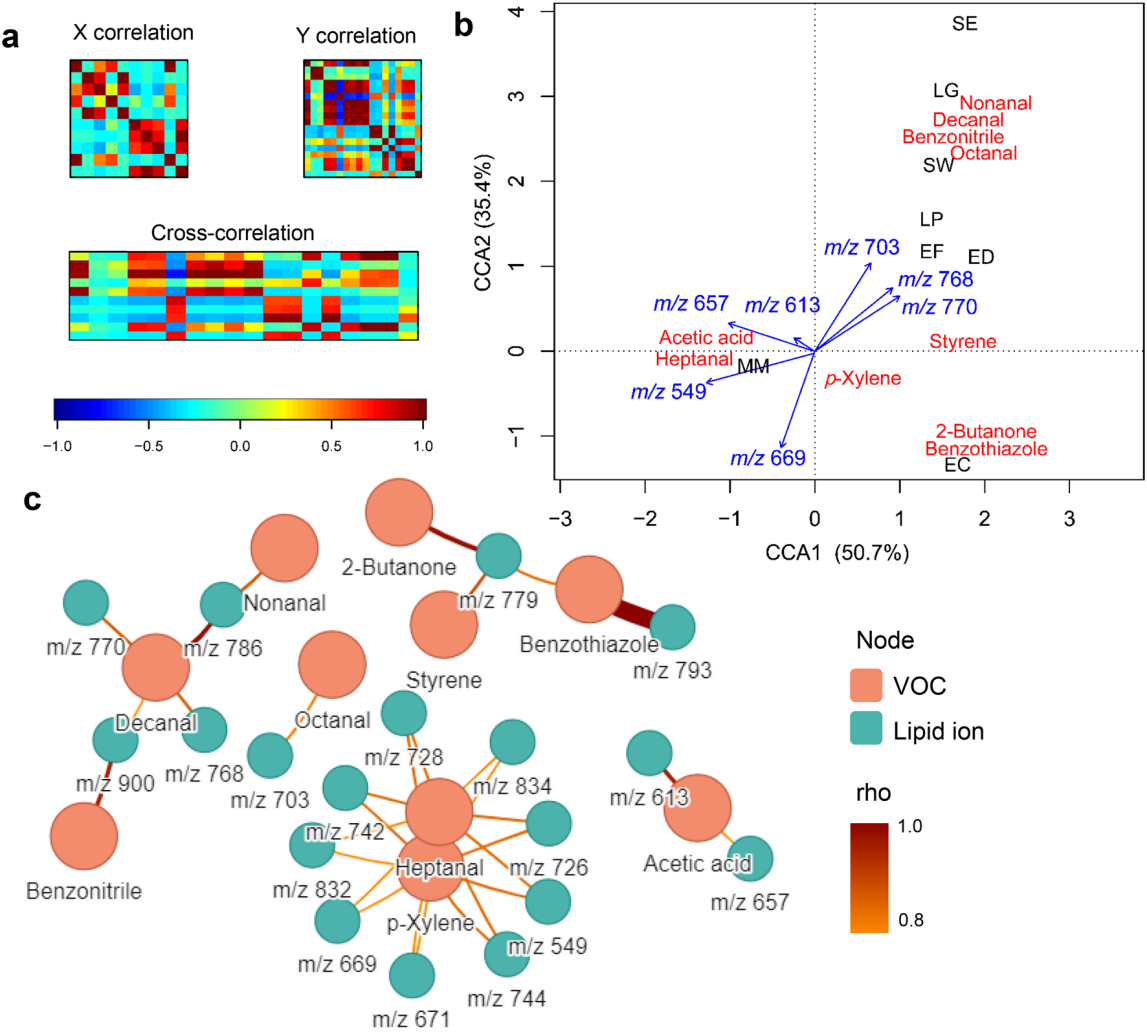
(**a**) Cross-correlation matrix (X–Y) obtained from combining the matrices X (VOCs) and Y (lipid ions in the chloroform fraction); (**b**) CCA biplot; (**c**) networks showing the significant associations between lipid ions and VOCs, according to Spearman correlation analysis (significance criteria: *p* < 0.05, rho =|0.8|). Edge color and thickness refers to the strength and significance (− Log_10_ *p*) of the correlation, respectively.

Methods of correlation analysis were used to investigate associations between lipids profiles recovered from the chloroform fraction, VOCs and bacterial species. Correlation maps presented in Fig. 6a provide an overview of the abundance and nature of all possible bicorrelations existing among VOCs (matrix X) and lipids (matrix Y). In these plots, dark blue hues denote VOCs or lipids displaying strong negative correlations, red hues refer to strong positive correlations, while cyan and green hues denote very weak/irrelevant correlations. A cross-correlation matrix (X–Y) obtained from the combination between VOCs and NALDI-MS data exhibits several strong correlations between volatiles and lipids, suggesting a relevant relationship between both datasets. Figure 6b summarizes CCA output, presenting a merging between results for site and variables scores. The orientation and length of arrows are proportional to the degree of correlation a lipid has with the VOCs and bacterial species. G− and G + bacteria were separated by the first component—a discrimination ruled mainly by the lipids at *m/z* 549 and 657, which are the most correlated with CCA1. In contrast, the ions at *m/z* 703, 768 and 770 are associated with G + bacteria and the production of nonanal, decanal, octanal and benzonitrile by them. In this line, the lipids at *m/z* 657 and 549 appear as strongly associated with the generation of acetic acid and heptanal, respectively.

Spearman correlation analysis allowed us to find connections between individual lipids and microbial VOCs, a network view of these results show 5 groups of relationships (Fig. 6c). The first one, related to the 2-butanone, styrene and benzothiazole; the second one, regarding heptanal and *p*-xylene; the third one, regarding acetic acid; the fourth one, showing the correspondences between nonanal, decanal and benzonitrile; lastly, the fifth one regards octanal trends. Overall, it showed an association between NALDI-MS lipid profiles and the metabolism of fatty acids (giving rise to the fatty aldehydes heptanal, octanal, nonanal and decanal), the metabolism of amino acids (benzonitrile and benzothiazole), fermentation pathways (acetic acid and 2-butanone) and the metabolism of aromatic hydrocarbons by microorganisms (*p*-xylene, styrene). Fatty aldehydes such as the mentioned ones can be derived from the oxidation of lipids from the cell membranes, or during fatty acid metabolism through the reduction of fatty acyl-ACP^39^. Considering this, fatty aldehydes are metabolites expected to be linked with the lipid composition of bacteria. Aromatic amino acids, which are precursors of benzonitrile (through the aldoxime–nitrile pathway) and possibly also of benzothiazole are required for lipid stabilization^39,49^. Therefore, the metabolism of aromatic amino acids may be an indicative of lipid synthesis and composition. It is known that bacteria are able to metabolize polyaromatic hydrocarbons (PAHs) from the environment. Parallelly, the switch of bacterial metabolism towards the catabolism of PAHs may lead to changes in fatty acid content or composition in bacteria^50^. Fermentative metabolic routes generate pyruvate. Pyruvate when further converted into malonyl-CoA becomes precursor in the biosynthesis of fatty acids, which can be incorporated into lipids^39^.

## Conclusion

The present study demonstrated specificities of the metabolic profiles for eight strains of bacteria with application of polypyrrole-MOF solid-phase microextraction fibers and plasmonic silver nanostructured substrates. Due to simultaneous application of innovative devices, comprehensive profiling based on volatile and non-volatile metabolites was carried out for the first time. The main hypothesis of the study was that presented devices would enable registration of metabolic profiles, which could be valuable for species differentiation.

Volatile profiles collected with utilization of PPy@ZIF-8 SPME fibers consisted of nitrogen-containing compounds, ketones, aldehydes, alcohols, organic acids, which probably can be explained by affinity of fiber coating towards polar analytes. This is in agreement with the structure of polypyrrole ring, where nitrogen introduces affinity towards nitrogen-containing compounds and provides unequal distribution of electrons creating a permanent dipole moment. Although prevalence of nitrogen-containing compounds in collected profiles were possibly not attributed to the affinity of nitrogen in the structure of polypyrrole ring towards analytes, the results suggest that PPy@ZIF-8 fibers could serve as a cost-efficient alternative for VOCs profiling, offering relatively low cost and rapid synthesis, as well as mechanical and thermal stability.

Principal component analysis revealed distinction between G+ and G− species for both volatile and non-volatile profiles. Hierarchical cluster analysis showed discrimination between G+ and G− species and six clusters based on volatile profiles. In case of non-volatile profiles of chloroform fraction of the B & D bacterial extract, eight clusters corresponding to each species were observed in addition to distinction between G+ and G− species. Random forest model showed high accuracy (100%, 95%CI [79.4, 100%]) for classification of species using chloroform fraction of B & D extract in contrast to the methanolic fraction (95%CI [47.6, 92.7%]).

In case of non-volatile profiling, clear discrimination between eight species was achieved, potentially attributed to enhanced sensitivity of the NALDI-MS substrates towards low molecular weight metabolites due to plasmonic properties of silver nanoparticles. Failure of discrimination in case of polar phase of bacterial extracts possibly could be attributed either by fragmentation of polar lipids or their low content. Fragmentation of polar lipids in NALDI-MS analysis with application of silver nanostructured substrates could be explained by the nature of the interactions between analytes and nanoparticles.

Furthermore, methods of correlation analysis suggested relationship between volatile and non-volatile datasets, which demonstrated that complementary application of presented lab-made devices could be useful for gaining valuable insights into bacterial metabolism.

### Supplementary Information

Supplementary Information.

## Data Availability

The datasets used and/or analyzed during the current study available from the corresponding author on reasonable request.

## References

[1] Maier T, Klepel S, Renner U, Kostrzewa M (2006). Fast and reliable MALDI-TOF MS-based microorganism identification. Nat. Methods.

[2] Mather CA, Rivera SF, Butler-Wu SM (2014). Comparison of the bruker biotyper and vitek MS matrix-assisted laser desorption ionization-time of flight mass spectrometry systems for identification of mycobacteria using simplified protein extraction protocols. J. Clin. Microbiol..

[3] Sohlenkamp C, Geiger O (2015). Bacterial membrane lipids: Diversity in structures and pathways. FEMS Microbiol. Rev..

[4] Leung LM (2017). Identification of the ESKAPE pathogens by mass spectrometric analysis of microbial membrane glycolipids. Sci. Rep..

[5] Liang T (2018). Rapid microbial identification and antibiotic resistance detection by mass spectrometric analysis of membrane lipids. Anal. Chem..

[6] Tanaka K (1988). Protein and polymer analyses up to *m/z* 100 000 by laser ionization time-of-flight mass spectrometry. Rapid Commun. Mass Spectrom..

[7] Abdelhamid HN (2018). Nanoparticle assisted laser desorption/ionization mass spectrometry for small molecule analytes. Microchim. Acta.

[8] Su H (2021). Plasmonic alloys reveal a distinct metabolic phenotype of early gastric cancer. Adv. Mater..

[9] Huang L (2017). Plasmonic silver nanoshells for drug and metabolite detection. Nat. Commun..

[10] Labows JN, McGinley KJ, Webster GF, Leyden JJ (1980). Headspace analysis of volatile metabolites of *Pseudomonas aeruginosa* and related species by gas chromatography–mass spectrometry. J. Clin. Microbiol..

[11] Chen J, Tang J, Shi H, Tang C, Zhang R (2017). Characteristics of volatile organic compounds produced from five pathogenic bacteria by headspace-solid phase micro-extraction/gas chromatography–mass spectrometry. J. Basic Microbiol..

[12] Arthur CL, Pawliszyn J (1990). Solid phase microextraction with thermal desorption using fused silica optical fibers. Anal. Chem..

[13] Reese KL, Rasley A, Avila JR, Jones D, Frank M (2020). Metabolic profiling of volatile organic compounds (VOCs) emitted by the pathogens *Francisella tularensis* and *Bacillus anthracis* in liquid culture. Sci. Rep..

[14] Fitzgerald S, Duffy E, Holland L, Morrin A (2020). Multi-strain volatile profiling of pathogenic and commensal cutaneous bacteria. Sci. Rep..

[15] Lashgari M, Yamini Y (2019). An overview of the most common lab-made coating materials in solid phase microextraction. Talanta.

[16] Wang W, Kang S, Vikesland PJ (2021). Surface-enhanced Raman spectroscopy of bacterial metabolites for bacterial growth monitoring and diagnosis of viral infection. Environ. Sci. Technol..

[17] Mametov R, Sagandykova G, Monedeiro-Milanowski M, Gabryś D, Pomastowski P (2023). Electropolymerized polypyrrole-MOF composite as a coating material for SPME fiber for extraction VOCs liberated by bacteria. Sci. Rep..

[18] Maslak E (2022). A new approach to imaging and rapid microbiome identification for prostate cancer patients undergoing radiotherapy. Biomedicines.

[19] Złoch M, Pomastowski P, Maślak E, Monedeiro F, Buszewski B (2020). Study on molecular profiles of *Staphylococcus aureus* strains: Spectrometric approach. Molecules.

[20] Bligh EG, Dyer WJ (1959). A rapid method for extraction and purification. Can. J. Biochem. Physiol..

[21] Cockerill III, F.R. *et al*. & Abstract. *Methods for Dilution Antimicrobial Susceptibility Tests for Bacteria that Grow Aerobically; Approved Standard—Ninth Edition*. Vol. 32 (2012).

[22] Sagandykova G (2022). Silver nanostructured substrates in LDI-MS of low molecular weight compounds. Materials (Basel)..

[23] Son MS, Taylor RK (2021). Growth and maintenance of *Escherichia coli* laboratory strains. Curr. Protoc..

[24] Amabilis-Sosa LE, Vazquez-Lopez M, Rojas GJL, Roe-Sosa A, Moeller-Chavez GE (2018). Efficient bacteria inactivation by ultrasound in municipal wastewater. Environments.

[25] Smetanková J (2012). Influence of aerobic and anaerobic conditions on the growth and metabolism of selected strains of *Lactobacillus plantarum*. Acta Chim. Slov..

[26] Mis Solval K, Chouljenko A, Chotiko A, Sathivel S (2019). Growth kinetics and lactic acid production of *Lactobacillus plantarum* NRRL B-4496, *L. acidophilus* NRRL B-4495, and *L. reuteri* B-14171 in media containing egg white hydrolysates. LWT.

[27] Emborg J, Dalgaard P, Ahrens P (2006). *Morganella psychrotolerans* sp. nov., a histamine-producing bacterium isolated from various seafoods. Int. J. Syst. Evol. Microbiol..

[28] Minnullina L, Kostennikova Z, Evtugin V, Akosah Y, Sharipova M (2022). Diversity in the swimming motility and flagellar regulon structure of uropathogenic *Morganella morganii* strains. Int. Microbiol..

[29] Mantripragada VP, Jayasuriya AC (2016). Effect of dual delivery of antibiotics (vancomycin and cefazolin) and BMP-7 from chitosan microparticles on *Staphylococcus epidermidis* and pre-osteoblasts in vitro. Mater. Sci. Eng. C.

[30] Zhang X (2012). Genome-wide identification of ampicillin resistance determinants in *Enterococcus faecium*. PLoS Genet..

[31] Xie X (2023). Prevalence, virulence, and antibiotics gene profiles in *Lactococcus garvieae* isolated from cows with clinical mastitis in China. Microorganisms.

[32] Baikuni A, Hawari FL, Ramadon D, Malik A (2023). Untargeted LC-QTOF-MS/MS based metabolomic profile approach of bacterial ferment lysates and skin commensal bacterial cocktail ferment lysates. Hayati J. Biosci..

[33] Milanowski M (2019). Profiling of VOCs released from different salivary bacteria treated with non-lethal concentrations of silver nitrate. Anal. Biochem..

[34] Drabińska N (2022). Application of a solid-phase microextraction-gas chromatography-mass spectrometry/metal oxide sensor system for detection of antibiotic susceptibility in urinary tract infection-causing *Escherichia coli—*A proof of principle study. Adv. Med. Sci..

[35] Pawliszyn, J. Theory of solid-phase microextraction. In *Handbook of Solid Phase Microextraction*. 13–59 10.1016/B978-0-12-416017-0.00002-4 (Elsevier Inc., 2012).

[36] Mametov R, Sagandykova G, Monedeiro F, Buszewski B (2021). Development of controlled film of polypyrrole for solid-phase microextraction fiber by electropolymerization. Talanta.

[37] Hu JB, Chen YC, Urban PL (2013). Coffee-ring effects in laser desorption/ionization mass spectrometry. Anal. Chim. Acta.

[38] Schulz S, Dickschat JS (2007). Bacterial volatiles: The smell of small organisms. Nat. Prod. Rep..

[39] Monedeiro F, Railean-Plugaru V, Monedeiro-Milanowski M, Pomastowski P, Buszewski B (2021). Metabolic profiling of vocs emitted by bacteria isolated from pressure ulcers and treated with different concentrations of bio-agnps. Int. J. Mol. Sci..

[40] Gosset G (2009). Production of aromatic compounds in bacteria. Curr. Opin. Biotechnol..

[41] Adams A, De Kimpe N (2007). Formation of pyrazines and 2-acetyl-1-pyrroline by *Bacillus cereus*. Food Chem..

[42] DeMilo AB, Lee CJ, Moreno DS, Martinez AJ (1996). Identification of volatiles derived from citrobacter freundii fermentation of a trypticase soy broth. J. Agric. Food Chem..

[43] Breil C, Abert Vian M, Zemb T, Kunz W, Chemat F (2017). “Bligh and Dyer” and Folch methods for solid–liquid–liquid extraction of lipids from microorganisms. Comprehension of solvatation mechanisms and towards substitution with alternative solvents. Int. J. Mol. Sci..

[44] Maslak E (2023). Silver nanoparticle targets fabricated using chemical vapor deposition method for differentiation of bacteria based on lipidomic profiles in laser desorption/ionization mass spectrometry. Antibiotics.

[45] Fahy E (2009). Update of the LIPID MAPS comprehensive classification system for lipids. J. Lipid Res..

[46] Oursel D (2007). Lipid composition of membranes of Escherichia coli by liquid chromatography/tandem mass spectrometry using negative electrospray ionization. Rapid Commun. Mass Spectrom..

[47] Li M, Yang L, Bai Y, Liu H (2014). Analytical methods in lipidomics and their applications. Anal. Chem..

[48] Jaber MA (2023). Advantages of using biologically generated ^13^C-labelled multiple internal standards for stable isotope-assisted LC–MS-based lipidomics. Anal. Methods.

[49] Mbaye MN (2019). A comprehensive computational study of amino acid interactions in membrane proteins. Sci. Rep..

[50] Seo JS, Keum YS, Li QX (2009). Bacterial degradation of aromatic compounds. Int. J. Environ. Res. Public Health.

