# Supplementary material for: Metabolic profiling of bacteria with the application of polypyrrole-MOF SPME fibers and plasmonic nanostructured LDI-MS substrates
Source: Sci Rep. 2024 Mar 6;14:5562. doi: 10.1038/s41598-024-56107-0 (PMC10917794; doi:10.1038/s41598-024-56107-0)
Supplement: Supplementary file 1 — Supplementary Information. [file 41598_2024_56107_MOESM1_ESM.docx]

***Electronic supplementary information***

**Metabolic Profiling of Bacteria with the Application of Polypyrrole-MOF SPME fibers and Plasmonic Nanostructured LDI-MS Substrates**

Radik Mametov ^a,§^, Gulyaim Sagandykova ^a^, Fernanda Monedeiro ^b^, Aleksandra Florkiewicz ^a^, Piotr Piszczek ^c^, Aleksandra Radtke ^c^ and Pawel Pomastowski ^a^

^a^ Centre for Modern Interdisciplinary Technologies, Nicolaus Copernicus University in Toruń, Wileńska 4, Toruń, 87-100, Poland

^b^ Department of Chemistry, Faculty of Philosophy, Sciences and Letters of Ribeirão Preto,

University of São Paulo, Av. Bandeirantes 3900, Ribeirão Preto, 14040-901, Brazil

^c^ Department of Inorganic and Coordination Chemistry, Faculty of Chemistry, Nicolaus Copernicus University in Toruń, Gagarina 7, Toruń, 87-100, Poland

^§^ corresponding author, e-mail: [mametov.radik@gmail.com](mailto:mametov.radik@gmail.com), tel. + 48 56 665 60 01

**Table of contents**

**Figure S1.** NALDI (dilution 1:10) and MALDI-TOF-MS (stock solution) spectra of the chloroform phase of the Bligh & Dyer extract of *Morganella morganii* collected by application of silver nanostructured substrates (NALDI), DHB and HCCA matrices (MALDI)

**Figure S2.** NALDI (dilution 1:10) and MALDI-TOF-MS (stock solution) spectra of the chloroform phase of the Bligh & Dyer extract of *Staphylococcus warneri* collected by application of silver nanostructured substrates (NALDI), DHB and HCCA matrices (MALDI)

**Figure S3.** NALDI (dilution 1:10) and MALDI-TOF-MS (stock solution) spectra of the chloroform phase of the Bligh & Dyer extract of *Lactobacillus plantarum* collected by application of silver nanostructured substrates (NALDI), DHB and HCCA matrices (MALDI)

**Figure S4.** NALDI (stock solution) and MALDI-TOF-MS (stock solution) spectra of the chloroform phase of the Bligh & Dyer extract of *Enterococcus faecium* collected by application of silver nanostructured substrates (NALDI), DHB and HCCA matrices (MALDI)

**Figure S5.** NALDI (dilution 1:10) and MALDI-TOF-MS (stock solution) spectra of the chloroform phase of the Bligh & Dyer extract of *Enterococcus durans* collected by application of silver nanostructured substrates (NALDI), DHB and HCCA matrices (MALDI)

**Figure S6.** NALDI (dilution 1:10) and MALDI-TOF-MS (stock solution) spectra of the chloroform phase of the Bligh & Dyer extract of *Lactococcus garvieae* collected by application of silver nanostructured substrates (NALDI), DHB and HCCA matrices (MALDI)

**Figure S7.** NALDI (dilution 1:10) and MALDI-TOF-MS (stock solution) spectra of the chloroform phase of the Bligh & Dyer extract of *Staphylococcus epidermidis* collected by application of silver nanostructured substrates (NALDI), DHB and HCCA matrices (MALDI)

**Figure S8.** NALDI (dilution 1:10) and MALDI-TOF-MS (stock solution) spectra of the chloroform phase of the Bligh & Dyer extract of *Escherichia coli* collected by application of silver nanostructured substrates (NALDI), DHB and HCCA matrices (MALDI)

**Figure S9.** NALDI and MALDI-TOF-MS (stock solution) spectra of the methanol fraction of the Bligh & Dyer extract of *Morganella morganii* collected by application of silver nanostructured substrates (NALDI), DHB and HCCA matrices (MALDI)

**Figure S10.** NALDI and MALDI-TOF-MS (stock solution) spectra of the methanol fraction of the Bligh & Dyer extract of *Staphylococcus warneri* collected by application of silver nanostructured substrates (NALDI), DHB and HCCA matrices (MALDI)

**Figure S11.** NALDI and MALDI-TOF-MS (stock solution) spectra of the methanol fraction of the Bligh & Dyer extract of *Lactobacillus plantarum* collected by application of silver nanostructured substrates (NALDI), DHB and HCCA matrices (MALDI)

**Figure S12.** NALDI and MALDI-TOF-MS (stock solution) spectra of the methanol phase of the Bligh & Dyer extract of *Enterococcus faecium* collected by application of silver nanostructured substrates (NALDI), DHB and HCCA matrices (MALDI)

**Figure S13.** NALDI and MALDI-TOF-MS (stock solution) spectra of the methanol fraction of the Bligh & Dyer extract of *Enterococcus durans* collected by application of silver nanostructured substrates (NALDI), DHB and HCCA matrices (MALDI)

**Figure S14.** NALDI and MALDI-TOF-MS (stock solution) spectra of the methanolic fraction of the Bligh & Dyer extract of *Lactococcus garvieae* collected by application of silver nanostructured substrates (NALDI), DHB and HCCA matrices (MALDI)

**Figure S15.** NALDI and MALDI-TOF-MS (stock solution) spectra of the methanolic fraction of the Bligh & Dyer extract of *Staphylococcus epidermidis* collected by application of silver nanostructured substrates (NALDI), DHB and HCCA matrices (MALDI)

**Figure S16.** NALDI and MALDI-TOF-MS (stock solution) spectra of the methanol fraction of the Bligh & Dyer extract of *Escherichia coli* collected by application of silver nanostructured substrates (NALDI), DHB and HCCA matrices (MALDI)

**FigureS17.** Microbial growth curves of bacteria grown at 37°C for 32h represented by optical density (OD) values: (a) *Escherichia coli*, (b) *Lactobacillus plantarum*, (c) *Morganella morganii*, (d) *Enterococcus durans*, (d) Control, Mueller Hinton Broth medium, (f) *Staphylococcus epidermidis*, (g) *Enterococcus faecium*, (h) *Lactococcus garvieae*, (i) *Staphylococcus warneri*

**Table S1.** Detected bacterial VOCs (after medium blanks subtraction) and their respective peak areas. Missing values refer to undetected peaks

**Table S2.** The list of *m/z* values and corresponding intensities for detected signals in chloroform fraction of B & D extract of *Morganella morganii* collected with NALDI-MS

**Table S3.** The list of *m/z* values and corresponding intensities for detected signals in chloroform fraction of B & D extract of *Staphylococcus warneri* collected with NALDI-MS

**Table S4.** The list of *m/z* values and corresponding intensities for detected signals in chloroform fraction of B & D extract of *Lactobacillus plantarum* collected with NALDI-MS

**Table S5.** The list of *m/z* values and corresponding intensities for detected signals in chloroform fraction of B & D extract of *Enterococcus faecium* collected with NALDI-MS

**Table S6.** The list of *m/z* values and corresponding intensities for detected signals in chloroform fraction of B & D extract of *Enterococcus durans* collected with NALDI-MS

**Table S7.** The list of *m/z* values and corresponding intensities for detected signals in chloroform fraction of B & D extract of *Lactococcus garvieae* collected with NALDI-MS

**Table S8.** The list of *m/z* values and corresponding intensities for detected signals in chloroform fraction of B & D extract of *Staphylococcus epidermidis* collected with NALDI-MS

**Table S9.** The list of *m/z* values and corresponding intensities for detected signals in chloroform fraction of B & D extract of *Escherichia coli* collected with NALDI-MS

**Table S10.** The list of *m/z* values and corresponding intensities for detected signals in methanol fraction of B & D extract of *Morganella morganii* collected with NALDI-MS

**Table S11.** The list of *m/z* values and corresponding intensities for detected signals in methanol fraction of B & D extract of *Staphylococcus warneri* collected with NALDI-MS

**Table S12.** The list of *m/z* values and corresponding intensities for detected signals in methanol fraction of B & D extract of *Lactobacillus plantarum* collected with NALDI-MS

**Table S13.** The list of *m/z* values and corresponding intensities for detected signals in methanol fraction of B & D extract of *Enterococcus faecium* collected with NALDI-MS

**Table S14.** The list of *m/z* values and corresponding intensities for detected signals in chloroform fraction of B & D extract of *Enterococcus durans* collected with NALDI-MS

**Table S15.** The list of *m/z* values and corresponding intensities for detected signals in methanol fraction of B & D extract of *Lactococcus garvieae* collected with NALDI-MS

**Table S16.** The list of *m/z* values and corresponding intensities for detected signals in methanol fraction of B & D extract of *Staphylococcus epidermidis* collected with NALDI-MS

**Table S17.** The list of *m/z* values and corresponding intensities for detected signals in methanol fraction of B & D extract of *Escherichia coli* collected with NALDI-MS

**Table S18.** Comparison of the extraction performance of PPY@ZIF-8 and CAR/PDMS SPME fibers for standard mixture of VOCs by HS-GC-FID analysis

**Figure S1.** NALDI (dilution 1:10) and MALDI-TOF-MS (stock solution) spectra of the chloroform phase of the Bligh & Dyer extract of *Morganella morganii* collected by application of silver nanostructured substrates (NALDI), DHB and HCCA matrices (MALDI)

**Figure S2.** NALDI (dilution 1:10) and MALDI-TOF-MS (stock solution) spectra of the chloroform phase of the Bligh & Dyer extract of *Staphylococcus warneri* collected by application of silver nanostructured substrates (NALDI), DHB and HCCA matrices (MALDI)

**Figure S3.** NALDI (dilution 1:10) and MALDI-TOF-MS (stock solution) spectra of the chloroform phase of the Bligh & Dyer extract of *Lactobacillus plantarum* collected by application of silver nanostructured substrates (NALDI), DHB and HCCA matrices (MALDI)

**Figure S4.** NALDI (stock solution) and MALDI-TOF-MS (stock solution) spectra of the chloroform phase of the Bligh & Dyer extract of *Enterococcus faecium* collected by application of silver nanostructured substrates (NALDI), DHB and HCCA matrices (MALDI)

**Figure S5.** NALDI (dilution 1:10) and MALDI-TOF-MS (stock solution) spectra of the chloroform phase of the Bligh & Dyer extract of *Enterococcus durans* collected by application of silver nanostructured substrates (NALDI), DHB and HCCA matrices (MALDI)

**Figure S6.** NALDI (dilution 1:10) and MALDI-TOF-MS (stock solution) spectra of the chloroform phase of the Bligh & Dyer extract of *Lactococcus garvieae* collected by application of silver nanostructured substrates (NALDI), DHB and HCCA matrices (MALDI)

**Figure S7.** NALDI (dilution 1:10) and MALDI-TOF-MS (stock solution) spectra of the chloroform phase of the Bligh & Dyer extract of *Staphylococcus epidermidis* collected by application of silver nanostructured substrates (NALDI), DHB and HCCA matrices (MALDI)

**Figure S8.** NALDI (dilution 1:10) and MALDI-TOF-MS (stock solution) spectra of the chloroform phase of the Bligh & Dyer extract of *Escherichia coli* collected by application of silver nanostructured substrates (NALDI), DHB and HCCA matrices (MALDI)

**Figure S9.** NALDI and MALDI-TOF-MS (stock solution) spectra of the methanol fraction of the Bligh & Dyer extract of *Morganella morganii* collected by application of silver nanostructured substrates (NALDI), DHB and HCCA matrices (MALDI)

**Figure S10.** NALDI and MALDI-TOF-MS (stock solution) spectra of the methanol fraction of the Bligh & Dyer extract of *Staphylococcus warneri* collected by application of silver nanostructured substrates (NALDI), DHB and HCCA matrices (MALDI)

**Figure S11.** NALDI and MALDI-TOF-MS (stock solution) spectra of the methanol fraction of the Bligh & Dyer extract of *Lactobacillus plantarum* collected by application of silver nanostructured substrates (NALDI), DHB and HCCA matrices (MALDI)

**Figure S12.** NALDI and MALDI-TOF-MS (stock solution) spectra of the methanol phase of the Bligh & Dyer extract of *Enterococcus faecium* collected by application of silver nanostructured substrates (NALDI), DHB and HCCA matrices (MALDI)

**Figure S13.** NALDI and MALDI-TOF-MS (stock solution) spectra of the methanol fraction of the Bligh & Dyer extract of *Enterococcus durans* collected by application of silver nanostructured substrates (NALDI), DHB and HCCA matrices (MALDI)

**Figure S14.** NALDI and MALDI-TOF-MS (stock solution) spectra of the methanolic fraction of the Bligh & Dyer extract of *Lactococcus garvieae* collected by application of silver nanostructured substrates (NALDI), DHB and HCCA matrices (MALDI)

**Figure S15.** NALDI and MALDI-TOF-MS (stock solution) spectra of the methanolic fraction of the Bligh & Dyer extract of *Staphylococcus epidermidis* collected by application of silver nanostructured substrates (NALDI), DHB and HCCA matrices (MALDI)

**Figure S16.** NALDI and MALDI-TOF-MS (stock solution) spectra of the methanol fraction of the Bligh & Dyer extract of *Escherichia coli* collected by application of silver nanostructured substrates (NALDI), DHB and HCCA matrices (MALDI


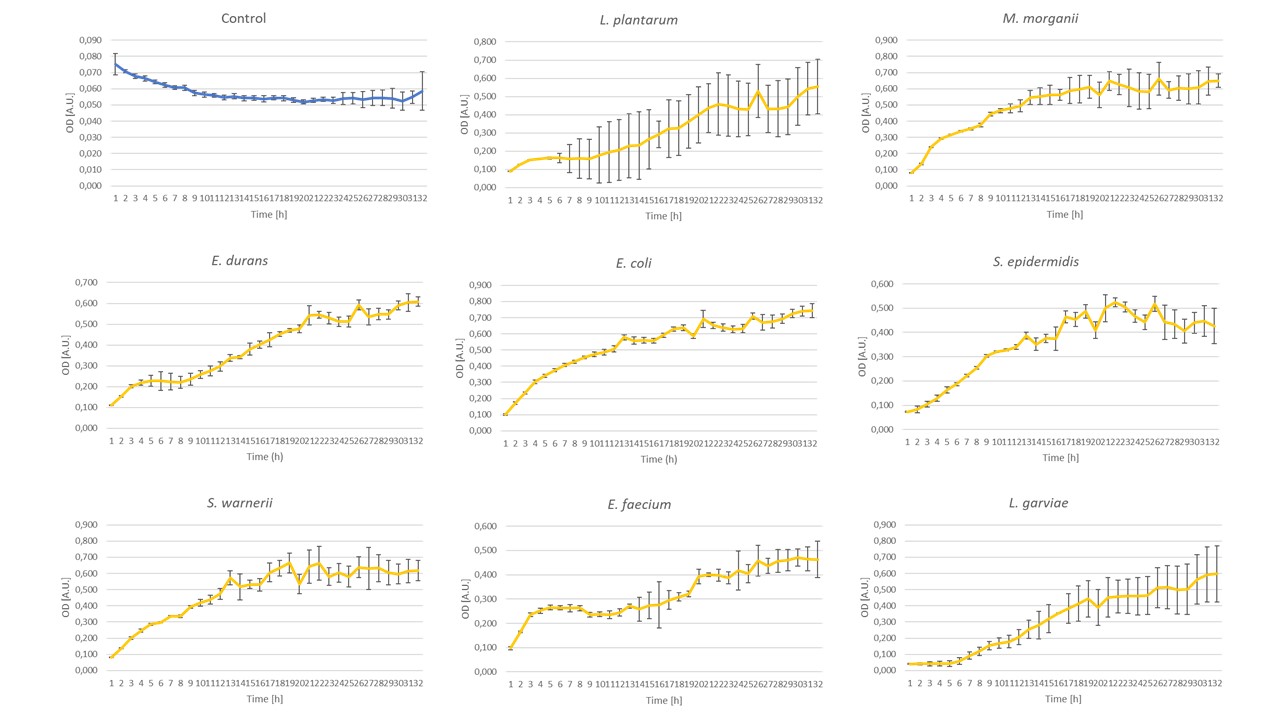


**FigureS17.** Microbial growth curves of bacteria grown at 37°C for 32h represented by optical density (OD) values: (a) *Escherichia coli*, (b) *Lactobacillus plantarum*, (c) *Morganella morganii*, (d) *Enterococcus durans*, (d) Control, Mueller Hinton Broth medium, (f) *Staphylococcus epidermidis*, (g) *Enterococcus faecium*, (h) *Lactococcus garvieae*, (i) *Staphylococcus warneri*

**Table S1.** Detected bacterial VOCs (after medium blanks subtraction) and their respective values of peak area. Missing values refer to undetected peaks and SE is Staphylococcus epidermidis, ED is Enterococcus durans, LP is Lactobacillus plantarum, SW is Staphylococcus Warneri, LG is Lactococcus garvieae, MM is Morganella morganii, EC is Escherichia coli, EF is Enterococcus Faecium

| Retention time (min) | Compound | *SE* | *ED* | *LP* | *SW* | *LG* | *MM* | *EC* | *EF* |
| --- | --- | --- | --- | --- | --- | --- | --- | --- | --- |
| 2,33 | 1,2-Propanediamine |  | 1138662,92 | 390867,33 |  |  |  |  |  |
| 3,31 | Dimethylamine |  |  | 4855489,4 | 1088816,2 |  |  |  |  |
| 3,34 | Nitro-ethane |  |  |  | 41586791 |  |  | 1509158,59 |  |
| 3,83 | Methylamine |  |  |  |  |  |  | 2426092 | 30346 |
| 6,28 | 2-Butanone |  | 426949,28 |  |  |  |  | 4014983,44 | 62352,25 |
| 7,04 | Ethyl acetate |  |  |  | 131275,2 |  |  | 183678,6 |  |
| 9,14 | Acetic acid |  |  |  |  |  | 1080942,21 |  | 12439,67 |
| 9,99 | Hexanal | 487786,08 | 436133,74 | 408817,52 | 1113320,7 |  |  | 415073,3 |  |
| 10,25 | Propanedioic acid | 388358,4 | 624676,71 | 290123,04 | 502056,79 | 819970,11 |  | 1381876,29 |  |
| 10,63 | Dimethyl disulfide |  |  |  |  |  | 72750645,8 | 350740,77 |  |
| 11,17 | 3-Methyl-1-butanol |  |  |  |  | 77276,77 | 5859607,22 |  |  |
| 11,88 | Oxalic acid |  | 179483,75 |  |  | 71725,44 |  | 431193,4 |  |
| 14,31 | 5-Hexen-2-ol |  | 578370,51 |  |  |  |  | 1659721,05 |  |
| 15,34 | 3-Hydroxybutanal |  | 150429,81 |  |  |  |  |  | 56443,19 |
| 15,42 | 3-Nitropropanoic acid |  |  |  |  | 44366,93 |  | 1453394,23 |  |
| 16,14 | p-Xylene |  |  |  |  |  | 1980426,01 | 1400289,51 | 101417,57 |
| 16,45 | Acetamide |  | 84658,63 |  |  | 137588,83 |  |  |  |
| 16,94 | Styrene |  | 461185,66 |  |  |  |  | 425513,91 | 40786,19 |
| 17,52 | Heptanal |  |  |  |  | 46680,89 | 33875190,9 | 119406,99 |  |
| 17,72 | 2,5-Dimethylpyrazine | 514852,74 | 393744,84 | 407965,28 | 959628,14 | 407882,64 |  | 439793,8 | 194395,47 |
| 19,45 | 1-Hexanamine |  |  |  | 96361,21 | 23677,82 |  |  |  |
| 20,08 | Benzaldehyde | 2349810,49 | 5568495,91 | 1707124,6 | 2487775,7 | 879167,45 | 704530,63 | 514533,83 |  |
| 20,62 | Octanal | 181531,24 | 97809,01 |  | 102107,05 |  |  |  | 14830,48 |
| 21,21 | Benzonitrile | 461082,24 |  |  |  | 127407,61 |  |  |  |
| 22,48 | Phenol | 486944,16 | 1076807,43 | 151849,19 | 2827457 | 2159439,09 | 117645838 | 8157424,19 | 273315,59 |
| 23,26 | Acetophenone | 174246,25 | 225291,87 | 144635,22 | 543144,99 |  |  |  |  |
| 23,47 | Nonanal | 336589,78 | 194583,36 |  |  | 244331,51 |  |  | 22670,02 |
| 24,85 | Benzothiazole |  |  |  |  |  |  | 2830557,79 | 51689,36 |
| 24,98 | Methyl-1-octadecanamine |  |  |  |  | 113586,18 |  |  | 24888,63 |
| 26,57 | Decanal | 503386,37 | 100028,04 |  |  | 124050,86 |  |  |  |
| 27,35 | Succinimide | 534652,17 | 2069917,2 | 516344,89 | 820908,3 | 1933478,72 | 87490432,8 |  |  |
| 28,94 | 2-Undecanone |  | 121503,92 |  |  | 317947,24 | 2354230,33 |  |  |
| 30,69 | Indole | 1893334,03 | 7784875,4 | 1834404,49 |  |  |  |  |  |
| 30,71 | 2-Dodecanone |  |  |  |  | 3081101,15 |  | 272264116,4 |  |
| 32,35 | 2-Tridecanone |  |  |  |  | 12423808 | 2525640,08 |  |  |
| 33,72 | 2-Tetradecanone | 433722,21 |  |  | 642101,14 | 2618168,27 | 2300326,35 |  |  |
| 34,34 | Tetracosane |  |  |  |  | 135990452 | 8308808,66 |  |  |
| 34,56 | Vinyl myristate | 604992,9 |  |  |  |  | 28315923,5 |  |  |
| 35,64 | Heptacosane |  |  |  | 426786,84 |  |  | 118562426,6 |  |

**Table S2**. The list of *m/z* values and corresponding intensities for detected signals in chloroform fraction of B & D extract of *Morganella morganii* collected with NALDI-MS

| rep 1 | | rep 2 | | rep 3 | | rep 4 | | rep 5 | |
| --- | --- | --- | --- | --- | --- | --- | --- | --- | --- |
| *m/z* | Intensity | *m/z* | Intensity | *m/z* | Intensity | *m/z* | Intensity | *m/z* | Intensity |
| 409 | 15255 | 409 | 12141 | 409 | 14759 | 409 | 20372 | 409 | 15242 |
| 425 | 22637 | 425 | 15145 | 425 | 23259 | 425 | 23622 | 425 | 23126 |
| 437 | 8747 | 437 | 8497 | 437 | 11115 | 437 | 13248 | 437 | 15301 |
| 439 | 5544 | 439 | 7472 | 439 | 7976 | 439 | 13024 | 439 | 10704 |
| 465 | 7812 | 465 | 6517 | 465 | 8030 | 465 | 14084 | 465 | 11359 |
| 467 | 6081 | 467 | 5188 | 467 | 8052 | 467 | 12302 | 467 | 11078 |
| 493 | 8740 | 493 | 5093 | 493 | 11448 | 493 | 15177 | 493 | 12111 |
| 495 | 7604 | 495 | 4161 | 495 | 8555 | 495 | 13766 | 495 | 9767 |
| 523 | 5709 | 549 | 4193 | 549 | 9421 | 549 | 11271 | 549 | 7297 |
| 549 | 11782 | 563 | 11873 | 563 | 28975 | 563 | 40410 | 563 | 29237 |
| 563 | 40012 | 669 | 5030 | 655 | 5961 | 655 | 8881 | 613 | 5232 |
| 655 | 5933 | 671 | 4372 | 657 | 6737 | 669 | 10859 | 655 | 7387 |
| 657 | 5717 | 685 | 16009 | 669 | 9174 | 671 | 10455 | 657 | 6013 |
| 669 | 8706 | 701 | 13495 | 671 | 8051 | 685 | 24510 | 669 | 9839 |
| 671 | 9162 | 702 | 5142 | 685 | 15130 | 701 | 20257 | 671 | 10341 |
| 685 | 15697 | 714 | 4714 | 700 | 5735 | 702 | 10532 | 674 | 5650 |
| 691 | 6196 | 726 | 9355 | 701 | 12558 | 707 | 9171 | 685 | 16915 |
| 701 | 19997 | 728 | 10466 | 702 | 6650 | 726 | 18653 | 701 | 19287 |
| 702 | 9686 | 730 | 5305 | 707 | 8795 | 728 | 22783 | 702 | 11579 |
| 707 | 9999 | 734 | 5235 | 714 | 6452 | 730 | 7977 | 707 | 8573 |
| 712 | 6136 | 742 | 40962 | 726 | 17056 | 734 | 8847 | 714 | 6283 |
| 714 | 6208 | 764 | 22338 | 728 | 19290 | 742 | 60788 | 726 | 16232 |
| 724 | 6956 | 769 | 8942 | 730 | 5672 | 744 | 6881 | 728 | 21379 |
| 726 | 17548 | 771 | 8772 | 734 | 5542 | 764 | 38854 | 730 | 8065 |
| 728 | 19471 | 773 | 4212 | 742 | 56239 | 769 | 18517 | 734 | 6076 |
| 730 | 7360 | 780 | 13391 | 744 | 7760 | 771 | 16852 | 742 | 67184 |
| 734 | 8518 | 795 | 4739 | 764 | 39588 | 773 | 6835 | 744 | 10205 |
| 742 | 71815 |  |  | 766 | 8615 | 780 | 23252 | 764 | 38814 |
| 764 | 45663 |  |  | 769 | 13290 | 782 | 6208 | 769 | 21426 |
| 769 | 10381 |  |  | 771 | 13019 | 795 | 6739 | 771 | 16489 |
| 771 | 10473 |  |  | 773 | 8195 | 811 | 6168 | 773 | 7463 |
| 773 | 6977 |  |  | 780 | 26557 | 832 | 6412 | 780 | 26105 |
| 778 | 5914 |  |  | 795 | 7965 |  |  | 795 | 6188 |
| 780 | 26214 |  |  | 811 | 5617 |  |  | 811 | 6002 |
| 795 | 7799 |  |  | 848 | 5374 |  |  | 834 | 5179 |
| 811 | 7556 |  |  |  |  |  |  | 848 | 5156 |
| 834 | 5119 |  |  |  |  |  |  | 850 | 5563 |
| 848 | 6058 |  |  |  |  |  |  |  |  |
| 850 | 6195 |  |  |  |  |  |  |  |  |

**Table S3.** The list of *m/z* values and corresponding intensities for detected signals in chloroform fraction of B & D extract of *Staphylococcus warneri* collected with NALDI-MS

| rep 1 | | rep 2 | | rep 3 | | rep 4 | | rep 5 | |
| --- | --- | --- | --- | --- | --- | --- | --- | --- | --- |
| *m/z* | Intensity | *m/z* | Intensity | *m/z* | Intensity | *m/z* | Intensity | *m/z* | Intensity |
| 409 | 72446 | 409 | 29859 | 409 | 31183 | 409 | 9771 | 409 | 43267 |
| 425 | 95707 | 425 | 44113 | 425 | 44400 | 425 | 10419 | 425 | 79895 |
| 437 | 7981 | 437 | 4144 | 437 | 11167 | 437 | 11030 | 437 | 7991 |
| 439 | 6265 | 467 | 5167 | 439 | 10312 | 439 | 9082 | 439 | 7047 |
| 465 | 8182 | 487 | 5402 | 444 | 5118 | 465 | 7527 | 444 | 6757 |
| 467 | 7655 | 493 | 10251 | 446 | 6089 | 467 | 8439 | 446 | 7309 |
| 487 | 8826 | 495 | 10418 | 465 | 12891 | 487 | 6057 | 465 | 7077 |
| 493 | 17652 | 551 | 4795 | 467 | 12033 | 493 | 11244 | 467 | 7803 |
| 495 | 14289 | 591 | 16006 | 487 | 5058 | 493 | 10258 | 487 | 5024 |
| 551 | 6375 | 607 | 5085 | 493 | 11082 | 495 | 9777 | 493 | 21858 |
| 563 | 11507 | 619 | 4757 | 495 | 14144 | 498 | 2728 | 495 | 18270 |
| 591 | 32218 | 675 | 5175 | 563 | 7072 | 551 | 10795 | 498 | 5760 |
| 605 | 9643 | 685 | 28658 | 591 | 25815 | 563 | 15064 | 551 | 5302 |
| 607 | 9271 | 701 | 32287 | 605 | 5930 | 591 | 15248 | 563 | 5969 |
| 619 | 11744 | 755 | 5210 | 607 | 6535 | 619 | 15784 | 591 | 19480 |
| 633 | 6067 | 761 | 11421 | 619 | 7434 | 685 | 15909 | 605 | 5734 |
| 675 | 8250 | 767 | 5610 | 675 | 8103 | 701 | 17186 | 607 | 8294 |
| 685 | 55548 | 769 | 9066 | 677 | 5803 | 761 | 14842 | 619 | 8496 |
| 701 | 61499 | 771 | 11505 | 685 | 57823 | 767 | 10646 | 675 | 6777 |
| 703 | 7566 | 783 | 10578 | 701 | 61379 | 769 | 16727 | 685 | 38706 |
| 733 | 9833 | 799 | 6315 | 733 | 6490 | 771 | 11945 | 701 | 53462 |
| 755 | 11238 | 887 | 10412 | 761 | 12905 | 783 | 16753 | 703 | 7773 |
| 761 | 21041 | 901 | 5374 | 767 | 5714 | 799 | 18454 | 733 | 5410 |
| 767 | 13788 | 903 | 8350 | 769 | 30658 | 887 | 20886 | 755 | 7136 |
| 769 | 12191 | 915 | 44051 | 771 | 28838 | 901 | 18010 | 761 | 14968 |
| 771 | 11987 | 929 | 7829 | 783 | 13513 | 903 | 22687 | 767 | 6712 |
| 783 | 24283 | 931 | 35419 | 799 | 9013 | 915 | 19117 | 769 | 19820 |
| 799 | 14423 | 943 | 6437 | 887 | 10037 | 929 | 22347 | 771 | 16938 |
| 887 | 16840 | 945 | 6483 | 903 | 10133 | 931 | 21091 | 783 | 18650 |
| 901 | 8030 | 957 | 3799 | 915 | 46170 | 943 | 11837 | 799 | 10473 |
| 903 | 14988 | 959 | 5666 | 929 | 7034 | 945 | 21045 | 887 | 10776 |
| 915 | 80565 | 999 | 3620 | 931 | 42577 | 959 | 20437 | 903 | 10533 |
| 929 | 14404 |  |  | 943 | 7246 | 1001 | 21770 | 915 | 49650 |
| 931 | 65983 |  |  | 945 | 7511 |  |  | 929 | 8602 |
| 943 | 13916 |  |  | 957 | 4237 |  |  | 931 | 48408 |
| 945 | 10479 |  |  | 959 | 6505 |  |  | 943 | 8738 |
| 957 | 8623 |  |  | 999 | 4775 |  |  | 945 | 8117 |
| 959 | 9148 |  |  | 1001 | 5057 |  |  | 957 | 4925 |
| 999 | 4748 |  |  |  |  |  |  | 959 | 8299 |
| 1199 | 4237 |  |  |  |  |  |  | 999 | 4145 |

**Table S4.** The list of *m/z* values and corresponding intensities for detected signals in chloroform fraction of B & D extract of *Lactobacillus plantarum* collected with NALDI-MS

| rep 1 | | rep 2 | | rep 3 | | rep 4 | | rep 5 | |
| --- | --- | --- | --- | --- | --- | --- | --- | --- | --- |
| *m/z* | Intensity | *m/z* | Intensity | *m/z* | Intensity | *m/z* | Intensity | *m/z* | Intensity |
| 409 | 55343 | 409 | 49483 | 409 | 46184 | 409 | 33846 | 409 | 11372 |
| 425 | 93785 | 425 | 101912 | 425 | 87453 | 425 | 71481 | 425 | 9476 |
| 437 | 13380 | 437 | 12567 | 437 | 15497 | 437 | 10205 | 437 | 4934 |
| 439 | 12932 | 439 | 11415 | 439 | 14127 | 439 | 11436 | 439 | 73343 |
| 444 | 8004 | 465 | 13141 | 465 | 17844 | 465 | 13742 | 465 | 11158 |
| 446 | 7477 | 467 | 11749 | 467 | 15961 | 467 | 11603 | 467 | 5567 |
| 465 | 14614 | 493 | 25814 | 493 | 25704 | 493 | 15484 | 493 | 9016 |
| 467 | 12278 | 495 | 22788 | 493 | 9171 | 493 | 5872 | 495 | 31007 |
| 493 | 19111 | 495 | 6393 | 495 | 23032 | 495 | 12264 | 495 | 5455 |
| 495 | 17392 | 591 | 6402 | 495 | 7409 | 495 | 5966 | 591 | 24641 |
| 563 | 7339 | 631 | 6529 | 617 | 10010 | 617 | 8738 | 617 | 6866 |
| 589 | 8711 | 685 | 65188 | 655 | 8494 | 685 | 51486 | 631 | 5225 |
| 591 | 9812 | 701 | 79142 | 685 | 56783 | 701 | 69291 | 655 | 51923 |
| 617 | 17993 | 769 | 34148 | 701 | 84156 | 703 | 8432 | 685 | 10770 |
| 631 | 11556 | 771 | 32726 | 769 | 43186 | 769 | 28276 | 701 | 90103 |
| 655 | 7960 | 885 | 26563 | 771 | 41130 | 771 | 27512 | 769 | 81802 |
| 657 | 6863 | 887 | 64274 | 785 | 7439 | 885 | 24005 | 771 | 11198 |
| 685 | 85184 | 901 | 20736 | 787 | 9324 | 887 | 50257 | 785 | 5322 |
| 701 | 105700 | 903 | 50440 | 885 | 26871 | 901 | 22896 | 805 | 5703 |
| 769 | 37255 | 911 | 19779 | 887 | 59983 | 903 | 43035 | 807 | 8715 |
| 771 | 34991 | 913 | 74046 | 901 | 21908 | 911 | 14329 | 885 | 6394 |
| 885 | 32810 | 915 | 28069 | 903 | 52112 | 913 | 60524 | 887 | 5461 |
| 887 | 64646 | 927 | 33636 | 911 | 19755 | 915 | 24305 | 901 | 6872 |
| 901 | 23559 | 929 | 52354 | 913 | 70745 | 927 | 26222 | 903 | 9331 |
| 903 | 49199 | 931 | 23320 | 915 | 27945 | 929 | 53230 | 911 | 11338 |
| 911 | 23997 | 937 | 6631 | 927 | 30686 | 931 | 19164 | 913 | 7163 |
| 913 | 78290 | 939 | 30106 | 929 | 57135 | 937 | 6807 | 915 | 10916 |
| 915 | 31321 | 941 | 59542 | 931 | 24948 | 939 | 26414 | 927 | 5992 |
| 927 | 38396 | 943 | 26507 | 939 | 33048 | 941 | 49601 | 929 | 14565 |
| 929 | 57816 | 945 | 6507 | 941 | 59701 | 943 | 22698 | 931 | 6402 |
| 931 | 25940 | 953 | 15040 | 943 | 27965 | 955 | 25551 | 937 | 90426 |
| 939 | 37515 | 955 | 27360 | 955 | 29122 | 957 | 43456 | 939 | 165590 |
| 941 | 66678 | 957 | 49723 | 957 | 54400 | 959 | 9988 | 941 | 81159 |
| 943 | 27373 | 959 | 11008 | 959 | 7657 | 960 | 7511 | 943 | 6080 |
| 955 | 32382 | 983 | 11597 | 983 | 9761 | 999 | 7927 | 953 | 8897 |
| 957 | 53621 | 985 | 7400 | 985 | 7104 | 1025 | 7029 | 955 | 9454 |
| 959 | 12630 | 997 | 7366 | 997 | 5962 |  |  | 957 | 6268 |
| 981 | 8028 | 999 | 7513 | 1025 | 8922 |  |  | 960 | 41620 |
| 983 | 12417 | 1025 | 7547 |  |  |  |  | 981 | 94935 |
| 985 | 8111 | 1027 | 6259 |  |  |  |  | 983 | 10129 |
| 997 | 7960 |  |  |  |  |  |  | 985 | 87934 |
| 999 | 7060 |  |  |  |  |  |  | 999 | 24008 |
|  |  |  |  |  |  |  |  | 1025 | 7545 |

**Table S5.** The list of *m/z* values and corresponding intensities for detected signals in chloroform fraction of B & D extract of *Enterococcus faecium* collected with NALDI-MS

| rep 1 | | rep 2 | | rep 3 | | rep 4 | | rep 5 | |
| --- | --- | --- | --- | --- | --- | --- | --- | --- | --- |
| *m/z* | Intensity | *m/z* | Intensity | *m/z* | Intensity | *m/z* | Intensity | *m/z* | Intensity |
| 409 | 18202 | 409 | 24039 | 409 | 30148 | 409 | 24386 | 409 | 33268 |
| 425 | 25499 | 425 | 35438 | 425 | 53131 | 425 | 30908 | 425 | 52911 |
| 437 | 26904 | 437 | 30985 | 437 | 27620 | 437 | 28908 | 437 | 31737 |
| 439 | 24822 | 439 | 26994 | 439 | 20826 | 439 | 26003 | 439 | 30395 |
| 444 | 8135 | 444 | 9074 | 465 | 25963 | 465 | 29446 | 446 | 9624 |
| 446 | 7986 | 465 | 29097 | 467 | 26518 | 467 | 23808 | 465 | 32623 |
| 465 | 28315 | 467 | 26324 | 493 | 13366 | 493 | 12133 | 467 | 31026 |
| 467 | 24832 | 493 | 13973 | 493 | 16154 | 493 | 16032 | 493 | 14505 |
| 493 | 10606 | 493 | 17479 | 495 | 13732 | 495 | 12281 | 493 | 19541 |
| 493 | 16626 | 495 | 15704 | 495 | 14749 | 495 | 15568 | 495 | 16212 |
| 495 | 15748 | 571 | 10937 | 611 | 7652 | 569 | 8032 | 495 | 18484 |
| 569 | 8064 | 573 | 7907 | 613 | 12836 | 611 | 9229 | 571 | 9167 |
| 571 | 8315 | 611 | 8170 | 615 | 9883 | 613 | 18503 | 611 | 11105 |
| 597 | 7101 | 613 | 15722 | 655 | 14866 | 615 | 13414 | 613 | 16584 |
| 611 | 10501 | 615 | 12420 | 657 | 11550 | 631 | 8756 | 615 | 10631 |
| 613 | 14255 | 655 | 18974 | 685 | 67652 | 639 | 8703 | 631 | 13658 |
| 631 | 7723 | 657 | 11491 | 697 | 11712 | 655 | 20720 | 655 | 20703 |
| 655 | 17520 | 685 | 62282 | 701 | 84838 | 657 | 14840 | 657 | 15188 |
| 657 | 11357 | 697 | 14859 | 739 | 11989 | 685 | 68564 | 685 | 89291 |
| 685 | 54792 | 699 | 9969 | 769 | 57533 | 697 | 17155 | 697 | 17792 |
| 697 | 15437 | 701 | 65527 | 771 | 54630 | 701 | 64037 | 699 | 13993 |
| 699 | 10453 | 703 | 9558 | 779 | 16830 | 703 | 9354 | 701 | 93276 |
| 701 | 50949 | 739 | 15309 | 783 | 7772 | 739 | 13718 | 739 | 13426 |
| 739 | 10452 | 769 | 59900 | 785 | 8956 | 741 | 10204 | 741 | 10280 |
| 741 | 7800 | 771 | 58411 | 793 | 9715 | 769 | 67395 | 769 | 81050 |
| 769 | 55185 | 779 | 13119 | 839 | 9997 | 771 | 67462 | 771 | 74076 |
| 771 | 47748 | 785 | 12211 | 873 | 11961 | 779 | 13255 | 779 | 16100 |
| 779 | 8799 | 787 | 10124 | 875 | 10371 | 785 | 12355 | 785 | 12317 |
| 781 | 7658 | 823 | 8933 | 887 | 7621 | 787 | 10289 | 787 | 14275 |
| 785 | 6698 | 873 | 12672 | 901 | 7151 | 823 | 9718 | 839 | 9164 |
| 873 | 12319 | 875 | 11176 | 903 | 7294 | 839 | 7957 | 873 | 15866 |
| 875 | 9212 | 911 | 9997 | 911 | 14034 | 873 | 12504 | 875 | 14597 |
| 911 | 7070 | 913 | 30558 | 913 | 42864 | 875 | 11705 | 885 | 8014 |
| 913 | 22881 | 915 | 10978 | 915 | 17867 | 887 | 7637 | 887 | 9571 |
| 915 | 8355 | 927 | 12570 | 927 | 17120 | 911 | 12193 | 911 | 16126 |
| 927 | 9109 | 929 | 24434 | 929 | 39262 | 913 | 35781 | 913 | 47839 |
| 929 | 18041 | 931 | 12081 | 931 | 21563 | 915 | 16539 | 915 | 22190 |
| 931 | 9143 | 939 | 33427 | 939 | 44461 | 927 | 15189 | 927 | 19228 |
| 939 | 25388 | 941 | 64549 | 941 | 80980 | 929 | 24744 | 929 | 41671 |
| 941 | 50894 | 955 | 31428 | 943 | 21057 | 931 | 14054 | 931 | 20332 |
| 953 | 10610 | 957 | 52490 | 955 | 42344 | 939 | 42022 | 939 | 51801 |
| 955 | 21829 | 959 | 14312 | 957 | 78142 | 941 | 72026 | 941 | 98775 |
| 957 | 39743 | 983 | 9918 | 959 | 18282 | 943 | 16206 | 955 | 46752 |
| 959 | 9306 | 1025 | 11433 | 983 | 14959 | 955 | 30821 | 957 | 87830 |
| 983 | 9244 | 1027 | 8483 | 1025 | 11841 | 957 | 51704 | 959 | 20469 |
| 1023 | 5303 |  |  | 1027 | 9947 | 959 | 13457 | 983 | 15671 |
| 1025 | 7143 |  |  | 1085 | 5875 | 983 | 11574 | 1025 | 13792 |
| 1027 | 6576 |  |  |  |  | 1025 | 10415 | 1027 | 12440 |
|  |  |  |  |  |  | 1027 | 8419 | 1069 | 6879 |

**Table S6.** The list of *m/z* values and corresponding intensities for detected signals in chloroform fraction of B & D extract of *Enterococcus durans* collected with NALDI-MS

| rep 1 | | rep 2 | | rep 3 | | rep 4 | | rep 5 | |
| --- | --- | --- | --- | --- | --- | --- | --- | --- | --- |
| *m/z* | Intensity | *m/z* | Intensity | *m/z* | Intensity | *m/z* | Intensity | *m/z* | Intensity |
| 409 | 13145 | 409 | 11066 | 409 | 9765 | 409 | 11793 | 409 | 8536 |
| 425 | 31201 | 425 | 21843 | 425 | 17177 | 425 | 29774 | 425 | 21807 |
| 437 | 43713 | 437 | 36393 | 437 | 25985 | 437 | 34577 | 437 | 36523 |
| 439 | 38908 | 439 | 31829 | 439 | 22678 | 439 | 29001 | 439 | 33127 |
| 444 | 10415 | 444 | 7789 | 444 | 7060 | 444 | 8823 | 444 | 9569 |
| 446 | 10049 | 465 | 32420 | 465 | 23060 | 446 | 8060 | 446 | 8357 |
| 465 | 39331 | 467 | 29893 | 467 | 22724 | 465 | 30726 | 465 | 32744 |
| 467 | 39376 | 493 | 8915 | 493 | 7100 | 467 | 27612 | 467 | 31108 |
| 493 | 10587 | 493 | 10155 | 495 | 6317 | 493 | 10644 | 493 | 8879 |
| 493 | 10922 | 495 | 8042 | 685 | 31058 | 493 | 7570 | 493 | 9331 |
| 495 | 11896 | 495 | 9024 | 701 | 47587 | 495 | 9459 | 495 | 8487 |
| 495 | 9345 | 685 | 44091 | 768 | 28512 | 495 | 7831 | 495 | 10223 |
| 685 | 48605 | 701 | 69987 | 770 | 27792 | 685 | 46593 | 685 | 35794 |
| 701 | 83153 | 703 | 10468 | 779 | 7105 | 701 | 86565 | 701 | 56940 |
| 768 | 55069 | 768 | 57492 | 912 | 20586 | 768 | 56118 | 703 | 9359 |
| 770 | 52832 | 770 | 52747 | 914 | 8410 | 770 | 49942 | 768 | 51293 |
| 779 | 12365 | 784 | 11570 | 926 | 7031 | 779 | 12467 | 770 | 46961 |
| 784 | 12720 | 786 | 11745 | 928 | 14537 | 784 | 12002 | 779 | 7527 |
| 786 | 9711 | 822 | 12204 | 930 | 7136 | 786 | 12186 | 784 | 9527 |
| 838 | 8090 | 838 | 8148 | 938 | 22605 | 822 | 11196 | 786 | 9532 |
| 872 | 12902 | 872 | 10146 | 940 | 46913 | 838 | 11911 | 822 | 7044 |
| 874 | 11663 | 874 | 7298 | 952 | 8353 | 872 | 10686 | 872 | 14165 |
| 910 | 8140 | 910 | 10509 | 954 | 19927 | 874 | 8573 | 874 | 13255 |
| 912 | 35910 | 912 | 40182 | 956 | 38335 | 910 | 10245 | 912 | 24836 |
| 914 | 14634 | 914 | 13823 | 958 | 7891 | 912 | 36566 | 914 | 9846 |
| 926 | 11521 | 926 | 10496 | 982 | 8987 | 914 | 14305 | 926 | 8191 |
| 928 | 34620 | 928 | 33869 | 1023 | 9348 | 926 | 10314 | 928 | 23785 |
| 930 | 16129 | 930 | 13697 | 1025 | 7217 | 928 | 33238 | 930 | 11149 |
| 938 | 37389 | 936 | 7980 |  |  | 930 | 15760 | 938 | 27113 |
| 940 | 83356 | 938 | 40108 |  |  | 938 | 39581 | 940 | 61325 |
| 952 | 12980 | 940 | 87612 |  |  | 940 | 82242 | 952 | 9455 |
| 954 | 39724 | 942 | 18310 |  |  | 954 | 38101 | 954 | 29394 |
| 956 | 78210 | 954 | 39976 |  |  | 956 | 82317 | 956 | 56595 |
| 958 | 18211 | 956 | 77141 |  |  | 958 | 18735 | 958 | 15530 |
| 982 | 16630 | 958 | 18608 |  |  | 982 | 18094 | 982 | 12700 |
| 997 | 7832 | 982 | 16110 |  |  | 1021 | 8207 | 997 | 6465 |
| 1021 | 8340 | 995 | 7756 |  |  | 1023 | 17901 | 1021 | 6173 |
| 1023 | 16446 | 997 | 7509 |  |  | 1025 | 13466 | 1023 | 15428 |
| 1025 | 13637 | 1021 | 10516 |  |  |  |  | 1025 | 10400 |
|  |  | 1023 | 19271 |  |  |  |  |  |  |
|  |  | 1025 | 16517 |  |  |  |  |  |  |

**Table S7**. The list of *m/z* values and corresponding intensities for detected signals in chloroform fraction of B & D extract of *Lactococcus garvieae* collected with NALDI-MS

| rep 1 | | rep 2 | | rep 3 | | rep 4 | | rep 5 | |
| --- | --- | --- | --- | --- | --- | --- | --- | --- | --- |
| m/z | Intensity | m/z | Intensity | m/z | Intensity | m/z | Intensity | m/z | Intensity |
| 409 | 11552 | 409 | 11900 | 409 | 8151 | 409 | 9071 | 409 | 6515 |
| 425 | 21053 | 425 | 30948 | 425 | 21085 | 425 | 16161 | 425 | 16572 |
| 437 | 27676 | 437 | 29283 | 437 | 26121 | 437 | 18753 | 437 | 19774 |
| 439 | 25846 | 439 | 26183 | 439 | 21511 | 439 | 16736 | 439 | 16434 |
| 465 | 28761 | 465 | 28489 | 465 | 25839 | 465 | 20049 | 465 | 19171 |
| 467 | 25440 | 467 | 27883 | 467 | 24464 | 467 | 16905 | 467 | 16580 |
| 493 | 8273 | 493 | 12070 | 493 | 7436 | 493 | 5417 | 493 | 4267 |
| 493 | 8547 | 495 | 9682 | 493 | 5476 | 493 | 4846 | 493 | 4603 |
| 495 | 7586 | 495 | 6022 | 495 | 6659 | 495 | 5249 | 495 | 5027 |
| 685 | 40074 | 685 | 43576 | 495 | 5266 | 685 | 39805 | 685 | 23765 |
| 701 | 52958 | 701 | 81846 | 685 | 39685 | 701 | 55734 | 701 | 40429 |
| 768 | 44001 | 703 | 9325 | 701 | 63205 | 768 | 31186 | 768 | 25214 |
| 770 | 41311 | 768 | 59432 | 768 | 48140 | 770 | 28038 | 770 | 24249 |
| 784 | 10767 | 770 | 52723 | 770 | 43356 | 784 | 7160 | 784 | 5574 |
| 786 | 8779 | 784 | 13064 | 784 | 10729 | 786 | 5566 | 786 | 5533 |
| 804 | 7288 | 786 | 11006 | 786 | 10753 | 804 | 8678 | 804 | 8263 |
| 806 | 9170 | 804 | 17579 | 804 | 13514 | 806 | 10579 | 806 | 9789 |
| 884 | 13111 | 806 | 16035 | 806 | 16194 | 822 | 5036 | 884 | 4729 |
| 886 | 37204 | 822 | 7756 | 822 | 5888 | 884 | 7389 | 886 | 18898 |
| 900 | 8451 | 838 | 6823 | 884 | 9801 | 886 | 23261 | 900 | 5970 |
| 902 | 26828 | 884 | 12919 | 886 | 32727 | 900 | 6410 | 902 | 17539 |
| 910 | 9613 | 886 | 35938 | 900 | 7849 | 902 | 22444 | 910 | 4863 |
| 912 | 39979 | 900 | 11406 | 902 | 26110 | 910 | 7365 | 912 | 17280 |
| 914 | 23976 | 902 | 41060 | 910 | 7388 | 912 | 25841 | 914 | 11078 |
| 924 | 7030 | 910 | 10264 | 912 | 31452 | 914 | 16129 | 926 | 14862 |
| 926 | 28614 | 912 | 38861 | 914 | 19987 | 926 | 19033 | 928 | 19301 |
| 928 | 30237 | 914 | 25480 | 926 | 25894 | 928 | 22345 | 930 | 11249 |
| 930 | 19765 | 926 | 32277 | 928 | 26436 | 930 | 12959 | 938 | 9280 |
| 938 | 18762 | 928 | 43440 | 930 | 18222 | 938 | 11798 | 940 | 25166 |
| 940 | 54711 | 930 | 27791 | 938 | 14375 | 940 | 35391 | 942 | 14402 |
| 942 | 24527 | 938 | 19014 | 940 | 43320 | 942 | 19690 | 952 | 4637 |
| 952 | 10381 | 940 | 54212 | 942 | 22628 | 952 | 7257 | 954 | 17612 |
| 954 | 32525 | 942 | 34484 | 952 | 7241 | 954 | 21002 | 956 | 26173 |
| 956 | 36493 | 952 | 8399 | 954 | 25552 | 956 | 29145 | 958 | 5931 |
| 958 | 8257 | 954 | 33283 | 956 | 37183 | 958 | 6801 | 980 | 7227 |
| 980 | 10453 | 956 | 55520 | 958 | 7345 | 980 | 8454 | 995 | 4223 |
| 982 | 9326 | 958 | 11141 | 980 | 9745 | 982 | 5505 | 1023 | 6050 |
| 984 | 6986 | 980 | 11209 | 982 | 6535 | 984 | 4599 |  |  |
| 995 | 6180 | 982 | 10872 | 983 | 5054 | 996 | 4707 |  |  |
| 997 | 6974 | 984 | 9309 | 985 | 5043 | 1023 | 5618 |  |  |
| 1023 | 7858 | 995 | 8786 | 995 | 6702 |  |  |  |  |
| 1025 | 6690 | 997 | 8616 | 997 | 8044 |  |  |  |  |
|  |  | 1023 | 11933 | 1023 | 9788 |  |  |  |  |
|  |  | 1025 | 10078 | 1025 | 7538 |  |  |  |  |
|  |  |  |  | 1037 | 4749 |  |  |  |  |

**Table S8**. The list of *m/z* values and corresponding intensities for detected signals in chloroform fraction of B & D extract of *Staphylococcus epidermidis* collected with NALDI-MS

| rep 1 | | rep 2 | | rep 3 | | rep 4 | | rep 5 | |
| --- | --- | --- | --- | --- | --- | --- | --- | --- | --- |
| *m/z* | Intensity | *m/z* | Intensity | *m/z* | Intensity | *m/z* | Intensity | *m/z* | Intensity |
| 409 | 33803 | 409 | 25940 | 409 | 27726 | 409 | 27032 | 409 | 23137 |
| 423 | 10297 | 425 | 41367 | 425 | 46072 | 425 | 43915 | 425 | 40448 |
| 425 | 46453 | 437 | 43853 | 437 | 32913 | 437 | 47914 | 437 | 45458 |
| 437 | 49745 | 439 | 38782 | 439 | 33495 | 439 | 44722 | 439 | 41038 |
| 439 | 49477 | 465 | 48768 | 451 | 7442 | 465 | 50378 | 465 | 48616 |
| 465 | 54800 | 467 | 40993 | 465 | 41986 | 467 | 52671 | 467 | 47238 |
| 467 | 49937 | 487 | 14993 | 467 | 34785 | 487 | 15740 | 487 | 12468 |
| 487 | 13769 | 493 | 14035 | 487 | 15293 | 493 | 18738 | 493 | 15309 |
| 493 | 17260 | 493 | 17062 | 493 | 15104 | 493 | 18111 | 493 | 14553 |
| 493 | 18678 | 495 | 14360 | 495 | 12147 | 495 | 16059 | 495 | 14280 |
| 495 | 16103 | 495 | 13394 | 498 | 15576 | 498 | 16824 | 495 | 13950 |
| 495 | 17751 | 498 | 16047 | 546 | 11374 | 546 | 12547 | 498 | 16262 |
| 498 | 14998 | 546 | 10309 | 551 | 12839 | 551 | 15815 | 546 | 9053 |
| 546 | 10421 | 551 | 16222 | 591 | 46355 | 565 | 10792 | 551 | 14556 |
| 551 | 16771 | 565 | 9443 | 605 | 26333 | 591 | 38237 | 565 | 8433 |
| 577 | 10481 | 591 | 34500 | 607 | 19903 | 605 | 21348 | 591 | 32596 |
| 591 | 56700 | 605 | 18487 | 619 | 21992 | 607 | 20503 | 605 | 18575 |
| 605 | 31726 | 607 | 13688 | 621 | 11721 | 619 | 21505 | 607 | 16467 |
| 607 | 23949 | 619 | 20917 | 633 | 14146 | 621 | 9183 | 619 | 18733 |
| 619 | 33639 | 633 | 12836 | 635 | 9249 | 633 | 14090 | 633 | 12044 |
| 621 | 11747 | 635 | 8563 | 675 | 13821 | 635 | 9586 | 675 | 14847 |
| 633 | 20437 | 675 | 14064 | 677 | 9924 | 675 | 15677 | 677 | 12702 |
| 635 | 11022 | 677 | 10458 | 685 | 82618 | 677 | 12016 | 685 | 74827 |
| 675 | 18509 | 685 | 75712 | 691 | 8900 | 685 | 77113 | 689 | 10749 |
| 677 | 16176 | 689 | 8692 | 701 | 92152 | 689 | 11425 | 701 | 89913 |
| 685 | 107484 | 701 | 88936 | 766 | 19630 | 701 | 91230 | 768 | 73610 |
| 689 | 12411 | 703 | 13598 | 768 | 51635 | 766 | 20260 | 770 | 66677 |
| 701 | 118949 | 766 | 17643 | 770 | 48368 | 768 | 68803 | 774 | 16128 |
| 703 | 18728 | 768 | 64215 | 774 | 17816 | 770 | 64997 | 782 | 31283 |
| 766 | 23418 | 770 | 64242 | 782 | 42068 | 774 | 19655 | 786 | 13984 |
| 768 | 71592 | 774 | 14888 | 788 | 11819 | 782 | 39661 | 788 | 13139 |
| 770 | 68483 | 782 | 34498 | 794 | 10576 | 786 | 13760 | 794 | 9742 |
| 774 | 20012 | 786 | 13515 | 796 | 24431 | 788 | 14785 | 796 | 18025 |
| 782 | 45405 | 788 | 10928 | 798 | 28695 | 794 | 11119 | 798 | 17964 |
| 786 | 14970 | 794 | 10359 | 810 | 19425 | 796 | 23049 | 810 | 14635 |
| 788 | 16021 | 796 | 20151 | 812 | 14871 | 798 | 26539 | 812 | 12502 |
| 794 | 15147 | 798 | 20576 | 824 | 14274 | 802 | 10454 | 824 | 12660 |
| 796 | 25397 | 810 | 16770 | 826 | 13454 | 810 | 18020 | 826 | 12103 |
| 798 | 26859 | 812 | 13052 | 840 | 8821 | 812 | 13224 | 900 | 8020 |
| 802 | 9803 | 824 | 12289 | 914 | 59723 | 824 | 13782 | 910 | 7519 |
| 810 | 19983 | 826 | 11556 | 928 | 35358 | 826 | 13698 | 914 | 42628 |
| 812 | 16855 | 840 | 8397 | 930 | 47315 | 840 | 9544 | 916 | 7977 |
| 824 | 17289 | 914 | 45738 | 942 | 33146 | 910 | 9197 | 928 | 29196 |
| 826 | 13966 | 916 | 8114 | 944 | 31458 | 914 | 55247 | 930 | 40365 |
| 840 | 11369 | 928 | 29670 | 956 | 29474 | 928 | 35771 | 942 | 25834 |
| 914 | 69207 | 930 | 43164 | 958 | 30464 | 930 | 54004 | 944 | 25969 |
| 916 | 10239 | 942 | 26314 |  |  | 942 | 33962 | 956 | 23483 |
| 928 | 47328 | 944 | 25655 |  |  | 944 | 36995 | 958 | 27490 |
| 930 | 59173 | 956 | 26661 |  |  | 956 | 27880 | 997 | 8210 |
| 942 | 46735 | 958 | 26964 |  |  | 958 | 29046 |  |  |
| 944 | 40303 | 997 | 6972 |  |  | 997 | 7930 |  |  |
| 956 | 35665 | 999 | 6549 |  |  |  |  |  |  |
| 958 | 38320 |  |  |  |  |  |  |  |  |
| 997 | 8689 |  |  |  |  |  |  |  |  |

**Table S9.** The list of *m/z* values and corresponding intensities for detected signals in chloroform fraction of B & D extract of *Escherichia coli* collected with NALDI-MS

| rep 1 | | rep 2 | | rep 3 | | rep 4 | | rep 5 | |
| --- | --- | --- | --- | --- | --- | --- | --- | --- | --- |
| *m/z* | Intensity | *m/z* | Intensity | *m/z* | Intensity | *m/z* | Intensity | *m/z* | Intensity |
| 437 | 20902 | 405 | 7428 | 405 | 5603 | 405 | 5646 | 403 | 5399 |
| 439 | 18634 | 409 | 9589 | 425 | 6981 | 437 | 27078 | 405 | 6173 |
| 465 | 20706 | 425 | 8878 | 437 | 29361 | 439 | 22976 | 425 | 5536 |
| 467 | 18168 | 437 | 39529 | 439 | 27902 | 465 | 23280 | 437 | 30394 |
| 563 | 22613 | 439 | 34406 | 444 | 5438 | 467 | 20886 | 439 | 28020 |
| 591 | 6221 | 465 | 35304 | 465 | 28273 | 563 | 21997 | 465 | 29316 |
| 669 | 6900 | 467 | 33374 | 467 | 28215 | 591 | 5265 | 467 | 24572 |
| 671 | 6830 | 538 | 7926 | 495 | 5533 | 669 | 8302 | 549 | 4945 |
| 685 | 6604 | 549 | 7665 | 563 | 24677 | 671 | 7959 | 563 | 24667 |
| 726 | 7662 | 563 | 41509 | 591 | 7780 | 726 | 5880 | 591 | 8508 |
| 728 | 5307 | 591 | 12373 | 669 | 10871 | 728 | 5432 | 655 | 5050 |
| 742 | 39200 | 669 | 17315 | 671 | 8144 | 742 | 25028 | 669 | 9464 |
| 763 | 21403 | 671 | 15503 | 685 | 10150 | 763 | 15194 | 671 | 7846 |
| 768 | 8138 | 685 | 20049 | 701 | 8178 | 768 | 9874 | 685 | 6963 |
| 770 | 10767 | 701 | 16841 | 706 | 6906 | 769 | 5723 | 701 | 6332 |
| 772 | 4570 | 706 | 12533 | 726 | 11115 | 770 | 9553 | 726 | 7080 |
| 779 | 15000 | 726 | 21328 | 728 | 10080 | 779 | 11999 | 728 | 6574 |
| 791 | 6033 | 728 | 15975 | 742 | 55196 | 791 | 4879 | 742 | 41768 |
| 807 | 4879 | 742 | 87468 | 763 | 35862 | 832 | 7636 | 763 | 28984 |
| 832 | 8623 | 744 | 9570 | 768 | 11354 | 834 | 9657 | 768 | 8825 |
| 834 | 11160 | 763 | 66641 | 769 | 13591 |  |  | 769 | 10069 |
| 847 | 5575 | 765 | 12227 | 770 | 12865 |  |  | 770 | 9006 |
| 849 | 4773 | 768 | 13240 | 777 | 6641 |  |  | 777 | 5056 |
|  |  | 770 | 24112 | 779 | 28476 |  |  | 779 | 24923 |
|  |  | 772 | 9876 | 791 | 9148 |  |  | 791 | 6883 |
|  |  | 775 | 11610 | 793 | 6852 |  |  | 793 | 6751 |
|  |  | 777 | 14854 | 807 | 8383 |  |  | 807 | 6513 |
|  |  | 779 | 48884 | 832 | 13846 |  |  | 832 | 10473 |
|  |  | 791 | 20629 | 834 | 17472 |  |  | 833 | 4562 |
|  |  | 794 | 9285 | 847 | 7037 |  |  | 834 | 11216 |
|  |  | 803 | 9062 | 849 | 7843 |  |  | 847 | 6382 |
|  |  | 807 | 15121 |  |  |  |  | 849 | 4807 |
|  |  | 810 | 10150 |  |  |  |  |  |  |
|  |  | 832 | 11976 |  |  |  |  |  |  |
|  |  | 834 | 16669 |  |  |  |  |  |  |
|  |  | 847 | 13883 |  |  |  |  |  |  |
|  |  | 849 | 12294 |  |  |  |  |  |  |

**Table S10.** The list of *m/z* values and corresponding intensities for detected signals in methanol fraction of B & D extract of *Morganella morganii* collected with NALDI-MS

| rep 1 | | rep 2 | | rep 3 | | rep 4 | | rep 5 | |
| --- | --- | --- | --- | --- | --- | --- | --- | --- | --- |
| *m/z* | Intensity | *m/z* | Intensity | *m/z* | Intensity | *m/z* | Intensity | *m/z* | Intensity |
| 409 | 35907 | 409 | 19983 | 409 | 54679 | 409 | 60802 | 409 | 51714 |
| 425 | 24723 | 425 | 21636 | 425 | 35760 | 425 | 57328 | 425 | 46491 |
| 437 | 12147 | 437 | 8928 | 437 | 15463 | 437 | 17701 | 437 | 18175 |
| 439 | 9919 | 439 | 6205 | 439 | 12724 | 439 | 16219 | 439 | 15946 |
| 465 | 12316 | 465 | 9226 | 465 | 18678 | 465 | 21616 | 465 | 16216 |
| 467 | 9820 | 467 | 8457 | 467 | 14414 | 467 | 17914 | 467 | 16483 |
| 493 | 15867 | 493 | 9084 | 493 | 25399 | 493 | 25281 | 493 | 22667 |
| 495 | 15687 | 495 | 7821 | 495 | 23442 | 495 | 26891 | 495 | 23232 |
| 764 | 7221 | 711 | 2898 | 708 | 7753 | 690 | 6203 | 674 | 6402 |
| 780 | 3712 | 764 | 4163 | 764 | 6727 | 708 | 7028 | 690 | 6718 |
|  |  | 779 | 3061 | 780 | 5899 | 764 | 13184 | 764 | 12203 |
|  |  | 795 | 2998 | 795 | 8277 | 779 | 7689 | 779 | 5133 |
|  |  |  |  | 811 | 5849 | 795 | 10835 | 780 | 6703 |
|  |  |  |  |  |  | 911 | 5987 | 795 | 8352 |
|  |  |  |  |  |  |  |  | 811 | 5887 |

**Table S11.** The list of *m/z* values and corresponding intensities for detected signals in methanol fraction of B & D extract of *Staphylococcus warneri* collected with NALDI-MS

| rep 1 | | rep 2 | | rep 3 | | rep 4 | | rep 5 | |
| --- | --- | --- | --- | --- | --- | --- | --- | --- | --- |
| *m/z* | Intensity | *m/z* | Intensity | *m/z* | Intensity | *m/z* | Intensity | *m/z* | Intensity |
| 409 | 51409 | 409 | 78883 | 409 | 53079 | 409 | 88017 | 409 | 74160 |
| 425 | 28831 | 425 | 44236 | 425 | 35921 | 425 | 52326 | 425 | 40805 |
| 465 | 8007 | 493 | 20103 | 437 | 7243 | 465 | 10245 | 437 | 9286 |
| 493 | 21171 | 495 | 17342 | 439 | 6746 | 467 | 9094 | 465 | 11758 |
| 495 | 19377 | 661 | 11804 | 465 | 9006 | 493 | 24039 | 467 | 11887 |
| 510 | 6694 | 689 | 8378 | 467 | 8940 | 495 | 23870 | 492 | 29864 |
| 512 | 8002 | 739 | 16392 | 493 | 19412 | 510 | 7637 | 494 | 26744 |
| 661 | 8877 | 745 | 11909 | 495 | 20419 | 605 | 8076 | 510 | 8339 |
| 689 | 6540 | 753 | 5275 | 605 | 5173 | 633 | 12111 | 512 | 6729 |
| 717 | 6484 | 755 | 14426 | 633 | 5786 | 661 | 27211 | 633 | 6788 |
| 739 | 7580 | 761 | 11855 | 661 | 15023 | 677 | 10770 | 661 | 14776 |
| 745 | 5334 | 767 | 40865 | 675 | 4556 | 689 | 19397 | 675 | 5301 |
| 755 | 5550 | 769 | 6475 | 677 | 5372 | 690 | 6444 | 689 | 10741 |
| 761 | 5788 | 781 | 5193 | 689 | 9942 | 705 | 6887 | 708 | 5500 |
| 767 | 17703 | 783 | 34634 | 705 | 4749 | 717 | 14043 | 710 | 5401 |
| 773 | 4476 | 797 | 5195 | 708 | 4666 | 733 | 7398 | 717 | 8352 |
| 783 | 12453 | 799 | 9421 | 717 | 8611 | 739 | 13159 | 733 | 7554 |
| 915 | 12328 | 887 | 5424 | 739 | 8643 | 745 | 9486 | 739 | 13167 |
| 931 | 4323 | 915 | 36287 | 745 | 7013 | 747 | 5540 | 745 | 10583 |
| 999 | 3089 | 929 | 7213 | 755 | 7364 | 755 | 10980 | 755 | 10883 |
|  |  | 931 | 12884 | 761 | 10259 | 761 | 15222 | 761 | 13059 |
|  |  | 943 | 5341 | 767 | 23266 | 767 | 37088 | 767 | 28749 |
|  |  |  |  | 773 | 4199 | 773 | 7802 | 769 | 4724 |
|  |  |  |  | 783 | 17715 | 781 | 5450 | 773 | 5822 |
|  |  |  |  | 799 | 4742 | 783 | 29710 | 783 | 20188 |
|  |  |  |  | 887 | 3774 | 799 | 9299 | 799 | 6230 |
|  |  |  |  | 915 | 22743 | 829 | 4630 | 887 | 5714 |
|  |  |  |  | 929 | 4028 | 887 | 6570 | 915 | 27506 |
|  |  |  |  | 931 | 8712 | 915 | 35863 | 929 | 3966 |
|  |  |  |  | 943 | 3114 | 929 | 7425 | 931 | 9941 |
|  |  |  |  | 999 | 3509 | 931 | 17447 | 999 | 4222 |
|  |  |  |  | 1001 | 3771 | 943 | 6541 | 1001 | 4216 |
|  |  |  |  |  |  | 999 | 4765 |  |  |

**Table S12.** The list of *m/z* values and corresponding intensities for detected signals in methanol fraction of B & D extract of *Lactobacillus plantarum* collected with NALDI-MS

| rep 1 | | rep 2 | | rep 3 | | rep 4 | | rep 5 | |
| --- | --- | --- | --- | --- | --- | --- | --- | --- | --- |
| *m/z* | Intensity | *m/z* | Intensity | *m/z* | Intensity | *m/z* | Intensity | *m/z* | Intensity |
| 409 | 106503 | 409 | 135851 | 409 | 73048 | 409 | 110951 | 409 | 136141 |
| 425 | 93073 | 425 | 126604 | 425 | 61014 | 425 | 105792 | 425 | 128261 |
| 493 | 33447 | 437 | 13060 | 492 | 19413 | 437 | 7636 | 492 | 41386 |
| 494 | 33664 | 439 | 12015 | 494 | 17865 | 444 | 8643 | 494 | 44776 |
| 527 | 167557 | 444 | 13828 | 527 | 200188 | 446 | 7648 | 527 | 199068 |
| 610 | 14065 | 446 | 14302 | 610 | 16989 | 465 | 11386 | 610 | 19456 |
| 612 | 11696 | 465 | 20450 | 612 | 15173 | 467 | 7629 | 612 | 18746 |
| 696 | 7952 | 467 | 17999 | 690 | 7595 | 492 | 54285 | 708 | 9900 |
| 710 | 7509 | 492 | 42058 | 696 | 8871 | 494 | 50467 | 710 | 8769 |
| 712 | 7555 | 494 | 40265 | 712 | 10840 | 527 | 107136 | 712 | 6903 |
| 764 | 19559 | 527 | 154273 | 748 | 7727 | 610 | 13223 | 734 | 7395 |
| 780 | 8944 | 610 | 14315 | 764 | 10219 | 612 | 10330 | 742 | 10957 |
| 887 | 7430 | 612 | 13187 | 780 | 6178 | 708 | 12182 | 764 | 33909 |
| 913 | 9395 | 675 | 10108 | 941 | 4948 | 710 | 9532 | 780 | 14713 |
| 927 | 4213 | 691 | 8184 |  |  | 726 | 4980 | 795 | 6238 |
| 939 | 5066 | 708 | 10890 |  |  | 742 | 7392 | 887 | 9096 |
| 941 | 11798 | 724 | 7510 |  |  | 764 | 22093 | 913 | 14273 |
| 955 | 4333 | 734 | 9147 |  |  | 780 | 10522 | 915 | 5564 |
|  |  | 742 | 14501 |  |  | 885 | 3911 | 927 | 4947 |
|  |  | 764 | 37789 |  |  | 887 | 8325 | 929 | 5669 |
|  |  | 780 | 16943 |  |  | 913 | 10834 | 939 | 11466 |
|  |  | 885 | 7371 |  |  | 939 | 6483 | 941 | 23439 |
|  |  | 887 | 14064 |  |  | 941 | 14687 | 955 | 7215 |
|  |  | 903 | 9110 |  |  | 955 | 5183 | 957 | 9658 |
|  |  | 913 | 20275 |  |  | 957 | 7025 |  |  |
|  |  | 915 | 7910 |  |  | 1027 | 3443 |  |  |
|  |  | 927 | 7088 |  |  |  |  |  |  |
|  |  | 929 | 8362 |  |  |  |  |  |  |
|  |  | 939 | 13887 |  |  |  |  |  |  |
|  |  | 941 | 25671 |  |  |  |  |  |  |
|  |  | 957 | 11396 |  |  |  |  |  |  |

**Table S13.** The list of *m/z* values and corresponding intensities for detected signals in methanol fraction of B & D extract of *Enterococcus faecium* collected with NALDI-MS

| rep 1 | | rep 2 | | rep 3 | | rep 4 | | rep 5 | |
| --- | --- | --- | --- | --- | --- | --- | --- | --- | --- |
| *m/z* | Intensity | *m/z* | Intensity | *m/z* | Intensity | *m/z* | Intensity | *m/z* | Intensity |
| 457 | 10271 | 444 | 6975 | 437 | 46874 | 437 | 31195 | 437 | 33735 |
| 527 | 14219 | 468 | 7633 | 444 | 116545 | 444 | 26543 | 441 | 13513 |
| 712 | 5029 | 685 | 5170 | 446 | 114185 | 446 | 23627 | 444 | 44497 |
|  |  | 696 | 5288 | 453 | 39281 | 453 | 19006 | 446 | 39277 |
|  |  | 941 | 7341 | 521 | 11902 | 527 | 20929 | 453 | 31434 |
|  |  |  |  | 527 | 16115 | 696 | 13522 | 457 | 12337 |
|  |  |  |  | 660 | 13943 | 712 | 10874 | 527 | 26273 |
|  |  |  |  | 662 | 13445 |  |  | 712 | 5731 |
|  |  |  |  | 712 | 8047 |  |  |  |  |
|  |  |  |  | 823 | 6038 |  |  |  |  |
|  |  |  |  | 913 | 5684 |  |  |  |  |
|  |  |  |  | 941 | 11113 |  |  |  |  |
|  |  |  |  | 955 | 4448 |  |  |  |  |
|  |  |  |  | 1133 | 3841 |  |  |  |  |
|  |  |  |  | 1199 | 4320 |  |  |  |  |

**Table S14.** The list of *m/z* values and corresponding intensities for detected signals in methanol fraction of B & D extract of *Enterococcus durans* collected with NALDI-MS

| rep 1 | | rep 2 | | rep 3 | | rep 4 | | rep 5 | |
| --- | --- | --- | --- | --- | --- | --- | --- | --- | --- |
| *m/z* | Intensity | *m/z* | Intensity | *m/z* | Intensity | *m/z* | Intensity | *m/z* | Intensity |
| 409 | 8294 | 409 | 6281 | 409 | 10595 | 409 | 9414 | 409 | 9083 |
| 437 | 7574 | 437 | 15237 | 437 | 16283 | 437 | 16620 | 437 | 22544 |
| 465 | 7825 | 439 | 12087 | 439 | 15418 | 439 | 12626 | 439 | 19240 |
| 467 | 7380 | 465 | 16513 | 465 | 18776 | 465 | 16259 | 465 | 22999 |
| 663 | 8475 | 467 | 14806 | 467 | 15982 | 467 | 17996 | 467 | 20035 |
| 679 | 6814 | 468 | 5684 | 663 | 8471 | 468 | 9971 | 468 | 5116 |
| 685 | 8037 | 470 | 5910 | 679 | 4438 | 470 | 8333 | 675 | 4161 |
| 696 | 6944 | 663 | 4602 | 685 | 7055 | 527 | 7169 | 680 | 3913 |
| 701 | 8288 | 682 | 3908 | 696 | 4403 | 663 | 8812 | 685 | 4916 |
| 940 | 3566 | 685 | 5040 | 701 | 5194 | 679 | 5692 | 912 | 1917 |
|  |  | 696 | 4879 | 746 | 4560 | 685 | 8435 | 940 | 3765 |
|  |  | 701 | 3870 | 940 | 3956 | 701 | 5914 |  |  |
|  |  | 940 | 2537 |  |  |  |  |  |  |

**Table S15.** The list of *m/z* values and corresponding intensities for detected signals in methanol fraction of B & D extract of *Lactococcus garvieae* collected with NALDI-MS

| rep 1 | | rep 2 | | rep 3 | | rep 4 | | rep 5 | |
| --- | --- | --- | --- | --- | --- | --- | --- | --- | --- |
| *m/z* | Intensity | *m/z* | Intensity | *m/z* | Intensity | *m/z* | Intensity | *m/z* | Intensity |
| 437 | 59208 | 437 | 58460 | 437 | 6709 | 437 | 6932 | 409 | 8508 |
| 439 | 56092 | 439 | 55040 | 441 | 6789 | 439 | 6116 | 437 | 13200 |
| 465 | 48531 | 465 | 60752 | 527 | 10075 | 441 | 7239 | 439 | 11419 |
| 467 | 42619 | 467 | 54359 | 711 | 4453 | 457 | 7133 | 441 | 8751 |
| 468 | 9762 | 591 | 11356 |  |  | 527 | 10252 | 457 | 11460 |
| 527 | 16824 | 619 | 10443 |  |  |  |  | 465 | 9949 |
| 652 | 11344 | 685 | 10839 |  |  |  |  | 467 | 7735 |
| 680 | 8242 | 711 | 9206 |  |  |  |  | 527 | 10045 |
| 685 | 7009 | 886 | 12693 |  |  |  |  |  |  |
| 768 | 6411 | 902 | 5412 |  |  |  |  |  |  |
| 770 | 7008 | 912 | 7681 |  |  |  |  |  |  |
| 902 | 3592 | 914 | 10142 |  |  |  |  |  |  |
| 912 | 3595 | 926 | 7907 |  |  |  |  |  |  |
| 940 | 9124 | 930 | 4190 |  |  |  |  |  |  |
| 954 | 4797 | 940 | 20422 |  |  |  |  |  |  |
| 956 | 3678 | 954 | 12638 |  |  |  |  |  |  |
|  |  | 956 | 8739 |  |  |  |  |  |  |
|  |  | 980 | 4218 |  |  |  |  |  |  |

**Table S16.** The list of *m/z* values and corresponding intensities for detected signals in methanol fraction of B & D extract of *Staphylococcus epidermidis* collected with NALDI-MS

| rep 1 | | rep 2 | | rep 3 | | rep 4 | | rep 5 | |
| --- | --- | --- | --- | --- | --- | --- | --- | --- | --- |
| *m/z* | Intensity | *m/z* | Intensity | *m/z* | Intensity | *m/z* | Intensity | *m/z* | Intensity |
| 437 | 32871 | 437 | 18819 | 437 | 32862 | 409 | 11056 | 409 | 19538 |
| 439 | 32627 | 439 | 15449 | 439 | 27315 | 437 | 107283 | 437 | 30864 |
| 465 | 37040 | 465 | 22562 | 465 | 36662 | 439 | 98788 | 439 | 29028 |
| 467 | 32055 | 467 | 18296 | 467 | 30061 | 446 | 9818 | 465 | 31370 |
| 682 | 4829 | 696 | 7218 | 574 | 8325 | 465 | 118675 | 467 | 30841 |
| 766 | 3613 | 714 | 6709 | 696 | 6258 | 467 | 118022 | 483 | 23516 |
|  |  | 766 | 6776 | 714 | 6283 | 468 | 10541 | 497 | 21431 |
|  |  |  |  | 766 | 5919 | 470 | 8931 | 499 | 20348 |
|  |  |  |  |  |  | 497 | 13874 | 574 | 23110 |
|  |  |  |  |  |  | 499 | 13603 | 590 | 14945 |
|  |  |  |  |  |  | 510 | 13368 | 674 | 11406 |
|  |  |  |  |  |  | 512 | 12057 | 692 | 15991 |
|  |  |  |  |  |  | 654 | 16658 | 695 | 17514 |
|  |  |  |  |  |  | 675 | 6758 | 711 | 11586 |
|  |  |  |  |  |  | 678 | 10100 | 714 | 21247 |
|  |  |  |  |  |  | 680 | 22602 |  |  |
|  |  |  |  |  |  | 682 | 20760 |  |  |
|  |  |  |  |  |  | 684 | 8273 |  |  |
|  |  |  |  |  |  | 766 | 14259 |  |  |
|  |  |  |  |  |  | 780 | 5900 |  |  |
|  |  |  |  |  |  | 782 | 8584 |  |  |
|  |  |  |  |  |  | 796 | 4132 |  |  |
|  |  |  |  |  |  | 914 | 6021 |  |  |

**Table S17.** The list of *m/z* values and corresponding intensities for detected signals in methanol fraction of B & D extract of *Escherichia coli* collected with NALDI-MS

| rep 1 | | rep 2 | | rep 3 | | rep 4 | | rep 5 | |
| --- | --- | --- | --- | --- | --- | --- | --- | --- | --- |
| *m/z* | Intensity | *m/z* | Intensity | *m/z* | Intensity | *m/z* | Intensity | *m/z* | Intensity |
| 404 | 20233 | 404 | 18550 | 404 | 20465 | 404 | 25221 | 404 | 24673 |
| 409 | 13787 | 409 | 13004 | 409 | 14996 | 409 | 17244 | 409 | 16682 |
| 437 | 64254 | 437 | 58447 | 437 | 51238 | 437 | 41357 | 437 | 47888 |
| 439 | 61676 | 439 | 50801 | 439 | 48164 | 439 | 35642 | 439 | 41877 |
| 465 | 71448 | 465 | 58174 | 465 | 58535 | 465 | 45660 | 465 | 54192 |
| 467 | 68722 | 467 | 52722 | 467 | 49930 | 467 | 39730 | 467 | 50249 |
| 487 | 12844 | 487 | 11445 | 654 | 9944 | 682 | 9531 | 680 | 10209 |
| 654 | 11429 | 654 | 9597 | 680 | 13160 | 696 | 10500 | 682 | 8969 |
| 680 | 15084 | 680 | 14225 | 682 | 12904 | 711 | 9913 | 696 | 10714 |
| 682 | 14507 | 682 | 12488 | 711 | 6841 | 763 | 15683 | 711 | 9636 |
| 711 | 8208 | 763 | 14135 | 763 | 16382 | 764 | 10416 | 763 | 12874 |
| 763 | 8022 | 764 | 8509 | 778 | 24609 | 778 | 28254 | 764 | 8899 |
| 764 | 7272 | 772 | 5831 | 780 | 10462 | 780 | 11704 | 778 | 22547 |
| 778 | 20175 | 778 | 26505 | 792 | 8118 | 792 | 10674 | 780 | 11381 |
| 780 | 7675 | 780 | 10802 | 794 | 28813 | 794 | 35108 | 791 | 7123 |
| 794 | 19792 | 792 | 7129 | 806 | 6615 | 806 | 7537 | 794 | 29765 |
| 808 | 6402 | 794 | 26832 | 808 | 5935 | 808 | 9052 | 806 | 6574 |
| 810 | 7764 | 806 | 6729 | 810 | 9558 | 810 | 11169 | 808 | 7627 |
| 822 | 6916 | 810 | 7645 | 822 | 7582 | 822 | 12310 | 810 | 9544 |
|  |  | 822 | 7226 |  |  |  |  | 822 | 9064 |

**Table S18.** Comparison of the extraction performance of PPY@ZIF-8 and CAR/PDMS SPME fibers for standard mixture of VOCs by HS-SPME-GC-FID analysis

|  | [PPy@ZIF-8](mailto:PPY@ZIF-8) | | | 75 μm CAR/PDMS | | |
| --- | --- | --- | --- | --- | --- | --- |
| Compound | Intensity (a. u.) | SD | RSD, % | Intensity (a. u.) | SD | RSD, % |
| Methanol | 1406.93 | 127.60 | 9.07 | 49.47 | 1.10 | 2.22 |
| Benzene | 93.17 | 10.10 | 10.84 | 185.93 | 10.24 | 5.51 |
| Toluene | 156.40 | 7.63 | 4.88 | 413.87 | 3.18 | 0.77 |
| Ethylbenzene | 262.23 | 9.94 | 3.79 | 576.17 | 38.32 | 6.65 |
| *p*-xylene | 434.30 | 17.82 | 4.10 | 589.23 | 8.04 | 1.36 |
| BFB | 377.13 | 28.15 | 7.46 | 1393.90 | 25.91 | 1.86 |
| Phenol | 224.40 | 12.40 | 5.53 | 69.63 | 2.80 | 4.02 |
| Dodecane | 1431.17 | 145.93 | 10.20 | 42.20 | 2.07 | 4.90 |
